# Comparative efficacy of non-pharmacological interventions for individuals with cannabis use disorder: a systematic review and network meta-analysis protocol

**DOI:** 10.1371/journal.pone.0355941

**Published:** 2026-08-21

**Authors:** Miaomiao Li, Deyu Miao, Qingcong Mo, Qinghua Chen, Liming Lu

**Affiliations:** 1 Medical College of Acu-Moxi and Rehabilitation, Guangzhou University of Chinese Medicine, Guangzhou, Guangdong, China; 2 South China Research Center for Acupuncture and Moxibustion, Guangzhou University of Chinese Medicine, Guangzhou, Guangdong, China; 3 The First School of Clinical medicine, Guangzhou University of Chinese Medicine, Guangzhou, Guangdon, China; UniEVANGELICA University Centre of Anapolis: UniEVANGELICA Centro Universitario de Anapolis, BRAZIL

## Abstract

**Background:**

Cannabis use disorder (CUD) imposes a significant global health burden, yet comparative evidence for the diverse range of non-pharmacological interventions (NPIs) remains fragmented. Existing reviews often combine diverse NPIs into broad categories, overlooking the specific contributions of neurobiological modalities. This study aims to establish a comprehensive comparative efficacy hierarchy for non-pharmacological CUD interventions by integrating psychosocial therapies and neurotherapy into a unified analytical framework.

**Methods:**

We will conduct a systematic review and network meta-analysis (NMA) of randomized controlled trials comparing NPIs against control group(including waitlist, no-intervention, or non-specific control) or another NPIs for individuals with CUD. Systematic searches will be performed across PubMed, Embase, the Cochrane Central Register of Controlled Trials (CENTRAL), and the Cumulative Index to Nursing and Allied Health Literature (CINAHL). Primary benefit outcomes will include biochemically verified cannabis abstinenc and self-reported days of cannabis use over the past 30 days. Primary acceptability will be evaluated by treatment retention, defined using an intention-to-treat approach as the proportion of randomized participants completing at least one session during the final treatment week, alongside follow-up retention rates. Primary safety will be assessed by the overall incidence of intervention-related adverse and serious adverse events. Pairs of reviewers will independently perform study selection, data extraction, and risk of bias. A frequentist NMA will be conducted to synthesize evidence. We will employ the Grading of Recommendations Assessment, Development and Evaluations(GRADE) approach to assess the certainty of evidence and categorize interventions using a minimally contextualized framework based on predefined decision thresholds.

**Results:**

Comprehensive search strategies have been validated across target databases. Formal study screening, data extraction, quantitative network meta-analyses, and GRADE evidence assessments are scheduled for completion by September 2026, followed by final manuscript preparation.

**Conclusion:**

This NMA will establish a comprehensive comparative evidence hierarchy for non-pharmacological CUD interventions by bridging psychosocial and neurobiological modalities. By pairing network estimates with GRADE certainty assessments, our findings will offer a standardized, actionable foundation for tailored clinical care and future trial methodology.

**Trial registration:**

PROSPERO CRD420251173215.

## Introduction

Cannabis use disorder (CUD) represents a major global public health burden. Recent Global Burden of Disease analyses identify cannabis as the most prevalent drug use disorder worldwide and a primary contributor to substance-related disability [1]. Epidemiological evidence indicates that 25% to 30% of cannabis users develop CUD, with a 2% to 3% annual prevalence among past-year users, particularly those engaging in frequent or high-potency use [2].

In North America, non-pharmacological interventions (NPIs) such as cognitive-behavioral therapy (CBT), motivational enhancement therapy (MET), and contingency management (CM) serve as the primary treatment options for CUD [3]. These behavioral therapies consistently reduce cannabis use frequency and dependence severity compared to inactive controls [4]. Mechanistically, CBT and MET enhance coping skills and readiness for change by reshaping learned associations between drug cues and rewards, whereas CM reinforces abstinence through tangible incentives that redirect reward learning toward non-drug outcomes [5–7]. In parallel, pharmacological options like synthetic cannabinoids and gabapentin have been explored, offering potential merits in mitigating acute withdrawal symptoms and craving; however, their clinical adoption remains constrained by key demerits, including modest overall efficacy, adverse effect risks, and a lack of formal regulatory approval [8]. Consequently, NPIs remain the frontline standard of care, though they are not universally curative, often require repeated sessions, and carry substantial resource demands [9].

Conventional systematic reviews of CUD treatments offer limited utility due to shared methodological constraints [10–14]. Aside from relying on small, localized samples, current frameworks inadequately explore heterogeneity and overlook effect modification driven by baseline clinical variations, thereby obscuring potential treatment-by-covariate interactions. Furthermore, most large-scale syntheses prioritize pharmacotherapies or broad psychosocial categories, effectively excluding specialized neurobiological modalities like acupuncture and repetitive Transcranial Magnetic Stimulation(rTMS). This oversight is critical because psychosocial interventions primarily target cognitive and behavioral patterns, while neuromodulatory modalities aim to directly modulate neural reward and stress circuits [7]. Whether these neuromodulatory approaches offer comparable or synergistic benefits to behavioral therapies remains an open question.

To address these critical gaps, we will conduct an systematic review and network meta-analysis (NMA) to evaluate both active-treatment and follow-up outcomes of non-pharmacological modalities, assessing the certainty of evidence with the Grading of Recommendations Assessment, Development and Evaluations(GRADE) approach. By integrating behavioral and neuromodulatory modalities into a unified comparative framework, this study will enable clinicians to match effective interventions with specific patient profiles and inform personalized clinical decision-making.

## Materials and methods

### Standardised reporting and registration

This systematic review and network meta-analysis protocols have been registered in PROSPERO (registration number: CRD420251173215) and reported in accordance with the Systematic Review and Meta-Analysis Protocols (PRISMA-P) 2015 guideline(S1 Table) [15].

### Search strategy

We preliminarily searched PubMed, Embase, the Cochrane Central Register of Controlled Trials (CENTRAL), and the Cumulative Index to Nursing and Allied Health Literature (CINAHL) from inception to December 30, 2025, without language restrictions. Prior to the final meta-analysis, the search will be fully updated to capture all newly published eligible studies. A methodologically trained researcher developed the search strategies, which were subsequently refined by a senior methodologist. Additionally, reference lists of eligible studies and relevant reviews will be manually screened to identify potential Randomized Controlled Trials(RCTs). The detailed search strategy is presented in S2 Table.

### Eligibility criteria

Table 1 presents the inclusion and exclusion criteria. We will include RCTs comparing any NPI against another NPI, treatment as usual (TAU), standard care, waiting list, or placebo. To account for clinical heterogeneity, participants will be classified into formally diagnosed CUD cases (DSM or ICD criteria) and screening-identified problematic cannabis users (e.g., recruited from detoxification or emergency settings, or reporting past-month use with baseline problems) [16].The primary analysis will synthesize all eligible populations combined. Pre-specified subgroup and sensitivity analyses will evaluate whether diagnostic certainty alters treatment estimates. If significant heterogeneity or subgroup differences are identified, findings will be stratified and reported separately by diagnostic certainty. Otherwise, the overall pooled synthesis will serve as the primary output.

**Table 1:** Eligibility criteria.

Domain	Eligibility criteria
Publication type	Original research reports
	Trial registrations for which no linked publication could be identified were eligible only if they provided outcome data.
	Conference abstracts, narrative reviews, comments, letters, or editorials were not eligible.
Study design	RCTs^a^
	Quasi-experimental trials, adaptive design, cross-over design, or secondary analysis of RCTs^a^ were not eligible.
Population	Adolescents (aged 12–17 years) and adults (aged ≥18 years) (on average or >50% of participants)
	Diagnosis of CUD^b^, or cannabis dependence or abuse, based on one of: **•** Recognized diagnostic criteria (e.g., any version of DSM^e^ or ICD^f^), **•** Individuals were recruited from a detoxification clinic, from emergency care visits for any reason, **•** Individuals who, at baseline, had both used cannabis in the past month and experienced at least one cannabis-related problem
	Excluded studies that enrolled patients who used cannabis for chronic non-cancer pain, cancer-related pain, and postoperative pain. Participants mandated to treatment by the criminal justice system were not eligible
Interventions	NPIs^d^ including: Psychological or psychotherapy interventions; Lifestyle modifications; Physical therapies; Complementary therapies; and Environmental and behavioural interventions. Individual or group-based
Comparators	NPIs^d^, TAU^c^, Waiting list, Delayed treatment control, No treatment, active control, or placebo, alone or in combination Comparisons between different formats of the same intervention were not eligible.
Outcomes	Primary outcomes (at the end of treatment and follow-up): 1)Primary benefit outcomes will include: (a) biochemically verified cannabis abstinence, and (b) total days of cannabis use over the past 30 days (self-reported). 2)Primary Acceptability Outcome: Treatment retention. 3)Primary Safety/Harm Outcome: Overall incidence of adverse events (and serious adverse events) attributed to the intervention. **Secondary outcomes(**at the end of treatment): 1)Quantity of cannabis use: Measured as the number of joints or grams consumed over the past 30 days. 2)Cannabis-related problems: Assessed via validated instruments such as the Cannabis Problems Scale. 3)Withdrawal and psychopathology: Withdrawal severity and associated psychological symptoms (including dependence, anxiety, and depression).
Timepoints	Pre-intervention (i.e., baseline) Post-intervention: further categorized into **•** Short-term treatment (≤4 weeks) **•** Medium-term treatment (≤12 weeks) **•** Long-term treatment (≤24 weeks) Follow-up **•** Short-term efficacy persistence (follow-up ≤24 weeks) **•** Long-term efficacy persistence (follow-up >24 weeks) Network meta-analysis was performed separately at each time point. If two or more follow-up assessments were available within the same time interval, the assessment closest to the lower bound of the respective category was selected.
Setting	Inpatient, outpatient or community-based treatment setting
Geography	No limitations
Publication language	No limitations

^**a**^randomized controlled trial; ^b^cannabis use disorder; ^c^Treatment as usual; ^d^non-pharmacological therapy; ^e^DSM = Diagnostic and Statistical Manual of Mental Disorders; ^f^ICD = International Classification of Diseases.

Age groups will be explicitly categorized as adolescents (aged 12–17 years) and adults (aged ≥18 years). To address potential clinical heterogeneity driven by age differences, age will be evaluated as a primary effect modifier across primary outcomes. Network meta-regressions incorporating trial-level mean age will be conducted to detect potential effect modification. Additionally, pre-specified subgroup analyses stratified by age category (adolescents vs. adults) and sensitivity analyses excluding adolescent trials will be performed to ensure the validity of the final network rankings.

Primary outcomes will evaluate intervention benefit, acceptability, and safety.

1) Primary benefit outcomes will include: (a) biochemically verified cannabis abstinence, and (b) total days of cannabis use over the past 30 days (self-reported).
2) Primary Acceptability Outcome: Treatment retention, defined as the proportion of randomized participants who complete at least one session during the final week of treatment, based on an intention-to-treat approach. Additionally, retention at follow-up timepoints will be calculated as the proportion of randomized participants completing follow-up assessments without dropout.
3) Primary Safety/Harm Outcome: Overall incidence of adverse events (and serious adverse events) attributed to the intervention.

Secondary outcomes will capture consumption intensity and broader clinical impacts, including:

1) Quantity of cannabis use: Measured as the number of joints or grams consumed over the past 30 days.
2) Cannabis-related problems: Assessed via validated instruments such as the Cannabis Problems Scale (CPS).
3) Withdrawal and psychopathology: Withdrawal severity and associated psychological symptoms (including dependence, anxiety, and depression).

Data will be extracted at baseline, post-intervention, and follow-up time points. Post-intervention outcomes will be categorized by treatment duration as short-term (≤4 weeks), medium-term (≤12 weeks), or long-term (≤24 weeks). Follow-up outcomes will be categorized by post-treatment efficacy persistence as short-term (≤24 weeks) or long-term (>24 weeks). Separate network meta-analyses will be conducted for each defined time horizon. If multiple assessments occur within the same time window, the evaluation closest to the category’s lower limit will be selected.

We will exclude studies that enrolled patients who used cannabis for chronic non-cancer pain, cancer-related pain, and postoperative pain, which may show systematically different responses to interventional procedures. Furthermore, the quasi-experimental, adaptive, and cross-over designs will be excluded to preserve internal validity and satisfy the transitivity assumption across the network. Specifically, cross-over trials carry substantial risks of persistent carryover effects in cognitive-behavioral and neuromodulatory interventions, while non-randomized designs introduce marked selection bias [17,18]. Although these strict restrictions minimize methodological heterogeneity, we acknowledge that excluding pragmatic or exploratory study designs may slightly limit the overall comprehensiveness of the retrieved trial landscape. The potential scope and implications of these exclusions will be formally discussed in the final review.

### Study selection

All identified citations will be exported to and managed using NoteExpress (v4.0.09855). Following the eligibility criteria, two trained reviewers will independently screen titles and abstracts in duplicate, followed by full-text evaluation of potentially eligible studies. Disagreements will be resolved through discussion or, if necessary, adjudicated by clinical experts blinded to trial results.

### Data extraction

Two investigators will independently extract data in duplicate using a standardized form (S3 Table), with disagreements resolved by consensus. We will extract study and participant characteristics, intervention details, and patient-reported outcomes. For continuous data, adjusted least-squares or baseline-adjusted end-scores will be prioritized, followed by change-from-baseline scores, and unadjusted end-scores. Outcomes describing monthly cannabis use will be standardized to a uniform 30-day timeframe (approximately 4.29 weeks). For multi-arm trials with a shared control, the control group sample size will be split proportionally across intervention comparisons to prevent double-counting participants [17].

We will extract the core components of each intervention and define them as network nodes, which will be further classified into first-level and second-level nodes. First-level nodes will represent commonly used single interventions as well as frequently applied two- or three-component combinations, thereby preserving network connectivity while avoiding excessive sparsity. These nodes encompassed specific behavioral, motivational, structural, neuromodulatory, and multi-component strategies (e.g., CBT, MET, CM, rTMS, transcranial direct current stimulation, or CBT + MET). Second-level nodes will provide a more granular representation of interventions, enabling sensitivity or subgroup analyses across different outcomes. Where applicable, second-level nodes will be prespecified for specific modalities (e.g., distinguishing abstinence-based CM from attendance-based CM).

Operational variations, including treatment intensity, duration, therapist contact, group versus individual setting, and face-to-face versus digital delivery, were systematically recorded. To prevent network disintegration into disconnected single-study nodes, these trial-level operational features were evaluated as potential sources of heterogeneity using pre-specified network meta-regressions and sensitivity analyses rather than serving as node-defining criteria.

### Risk of bias assessment

Pairs of reviewers will independently assess the risk of bias for each eligible study using the ROBUST-RCT instrument [19]. ROBUST-RCT evaluates bias across 14 domains, including six core and eight supplementary domains. Core domains address fundamental threats to internal validity: random sequence generation, allocation concealment, participant blinding, provider blinding, outcome assessor blinding, and incomplete outcome data. Supplementary domains evaluate baseline prognostic imbalance, co-intervention imbalance in blinded trials, group differences in outcome assessment, differential follow-up, validity of outcome measurements, as-treated analysis concerns, selective outcome reporting, and early termination for benefit.

Each domain will be rated as “definitely low,” “probably low” (low risk), “probably high,” or “definitely high” (high risk). Overall risk of bias will be determined a priori using predefined decision rules. For subjective outcomes, overall risk of bias will be rated as high if provider blinding, outcome assessor blinding, or incomplete outcome data is judged as high risk. For objective outcomes, overall risk of bias will be rated as high if sequence generation, allocation concealment, or selective outcome reporting is judged as high risk. Disagreements will be resolved by consensus or consultation with a third reviewer. Risk of bias judgments will be integrated into the interpretation of both direct and indirect NMA evidence.

### Data synthesis and statistical analysis

When mean change and standard deviations(SDs) were reported, we will utilize these directly. For studies providing pre- and post-intervention measures, we will apply the Cochrane Handbook methods to compute mean change and the standard deviations of change. If SDs were absent, we derived them from standard errors, P-values, confidence intervals(CIs), or graphical data [17]. Eligible studies that did not report patient-important outcomes or reported no data for quantitative analysis were included in our review, but did not contribute to meta-analyses.

Frequentist network meta-analyses will be performed using R (version 4.4.3). Treatment effects will be synthesized using random-effects NMA models, with between-study variance estimated via restricted maximum likelihood. Pairwise direct effects will be pooled when at least two trials address the same comparison. To assess local inconsistency between direct and indirect evidence, we will employ node-splitting models. Heterogeneity will be evaluated using the I^2^ statistic and the Chi^2^ test, in accordance with the Cochrane Handbook. Interpretation of heterogeneity will account for effect magnitude and direction alongside the strength of statistical evidence (e.g., P values and CIs). Publication bias will be assessed using funnel plots and Egger’s test if at least ten studies are available for a given outcome. All statistical tests will be two-tailed, with significance set at P ≤ 0.05.

Dichotomous outcomes will be pooled as Risk ratios(RR) with 95% CIs. For standardized and consistently defined binary safety metrics, including the incidence of total adverse events, serious adverse events, or treatment discontinuation attributed to adverse events, quantitative network synthesis will be performed, with pooled effect estimates expressed as RRs with 95% CIs. For specific, narrative, or heterogeneously reported adverse events across primary studies, qualitative descriptive synthesis will be conducted.

To accommodate potential variations in scoring metrics and assessment tools across included trials, continuous outcomes will be standardized prior to synthesis using Standardized Mean Differences (SMD) with 95% CIs. Scale orientations will be systematically aligned so that higher scores will consistently represent greater symptom severity or functional impairment across all studies. Beyond metric standardization, measurement tools will be audited to ensure they measure clinically comparable constructs. Potential residual heterogeneity originating from scale variations will be systematically evaluated using random-effects models, network meta-regressions, and pre-specified sensitivity analyses omitting outlying assessment instruments.

Absolute effect estimates will be calculated for dichotomous outcomes by applying pooled network relative estimates (RR) to the baseline risk of the control group, expressed as absolute risk differences per 1,000 participants over the treatment period.To evaluate imprecision for absolute outcomes, an absolute difference boundary of 50 events per 1,000 participants (5%) will serve as the decision threshold for clinical importance.

To evaluate potential effect modifiers, we will perform network meta-regressions under a common-coefficient assumption for each primary outcome, modeling log RRs for abstinence and treatment retention, and MDs for cannabis-use days. An identical set of modifiers will be evaluated across all three outcomes, including continuous variables (mean age, proportion of male participants, actual intervention duration, and follow-up length) and categorical variables (delivery format: digital/remote versus in-person; contingency management targets: adherence-based versus abstinence-based reinforcement). Regression coefficients, standard errors, and interaction P values will be reported. Robustness will be assessed through sensitivity analyses excluding trials with dropout rates exceeding 20% or those focusing on adolescent populations. In addition, prespecified subgroup analyses will be stratified by intervention duration and follow-up periods. All statistical tests will be two-tailed, with significance set at P < 0.05.

### Categorisation of interventions

We will rank interventions from most to least effective using a minimally contextualised approach, considering both effect estimates and the certainty of evidence [20,21]. Interventions will be classified into three decision categories based on effect estimates and evidence certainty: Category 1 (Least effective/acceptable): NPIs equivalent to control; Category 2: NPIs superior to control but not as effective as Category 3; Category 3: NPIs superior to at least one Category 2 intervention.

Superiority between intervention categories will be determined by combining point estimates against pre-specified decision thresholds (RR or OR ≥ 1.25 or ≤ 0.80; SMD ≥ 0.20 or ≤ −0.20) with the GRADE certainty of evidence [22,23]. “Superiority” does not strictly require the 95% CI to exclude the null effect value (1.0 or 0). Instead, an intervention will be classified as superior if its point estimate exceeds the clinical decision threshold and is supported by moderate or high certainty of evidence. Comparisons yielding low or very low certainty due to severe imprecision will be categorized as equivalent or uncertain regardless of point estimates.

### Certainty of the evidence

The certainty of evidence for direct, indirect, and network estimates will be evaluated using the GRADE framework for network meta-analyses [20]. Direct estimates from RCTs will initially start as high certainty and may be downgraded across five standard domains: risk of bias, inconsistency, indirectness, imprecision, and publication bias.

Certainty for indirect estimates will be derived from the lowest rating among the direct comparisons contributing the greatest weight to the dominant first-order loop (or higher-order loop if first-order loops are absent), as identified via a contribution matrix. Indirect ratings will be further downgraded if intransitivity is detected through clinical heterogeneity or an imbalanced distribution of effect modifiers across comparisons [24].

The overall network certainty will reflect the higher rating of its contributing direct and indirect estimates, with additional downgrading applied for inconsistency or imprecision between direct and indirect evidence. Imprecision will be rated based on whether 95% CIs cross the null effect for dichotomous outcomes or half of the minimally clinically important difference for continuous outcomes. In cases of severe inconsistency, the highest-certainty estimate (direct or indirect) will be prioritized.

Imprecision will be evaluated based on outcome type. For dichotomous outcomes, estimates will be downgraded if the total sample size fails to meet the optimal information size criterion or if 95% CIs cross the null effect. For continuous outcomes, imprecision will be rated down if 95% CIs encompass half of the minimal clinically important difference or cross thresholds for opposing clinical decisions.When inconsistency in random-effects models contributes to imprecision, the same effect estimate will not be downgraded twice. Outcomes informed by a single small trial with a large effect size will not be classified as high risk of bias; instead, they will be downgraded for imprecision or small-study bias during GRADE evaluation.

### Declaration of interests

The authors declare that they have no competing interests. The funders had no role in study design, data collection and analysis, decision to publish, or preparation of the manuscript.

## Results

**Fig 1:**
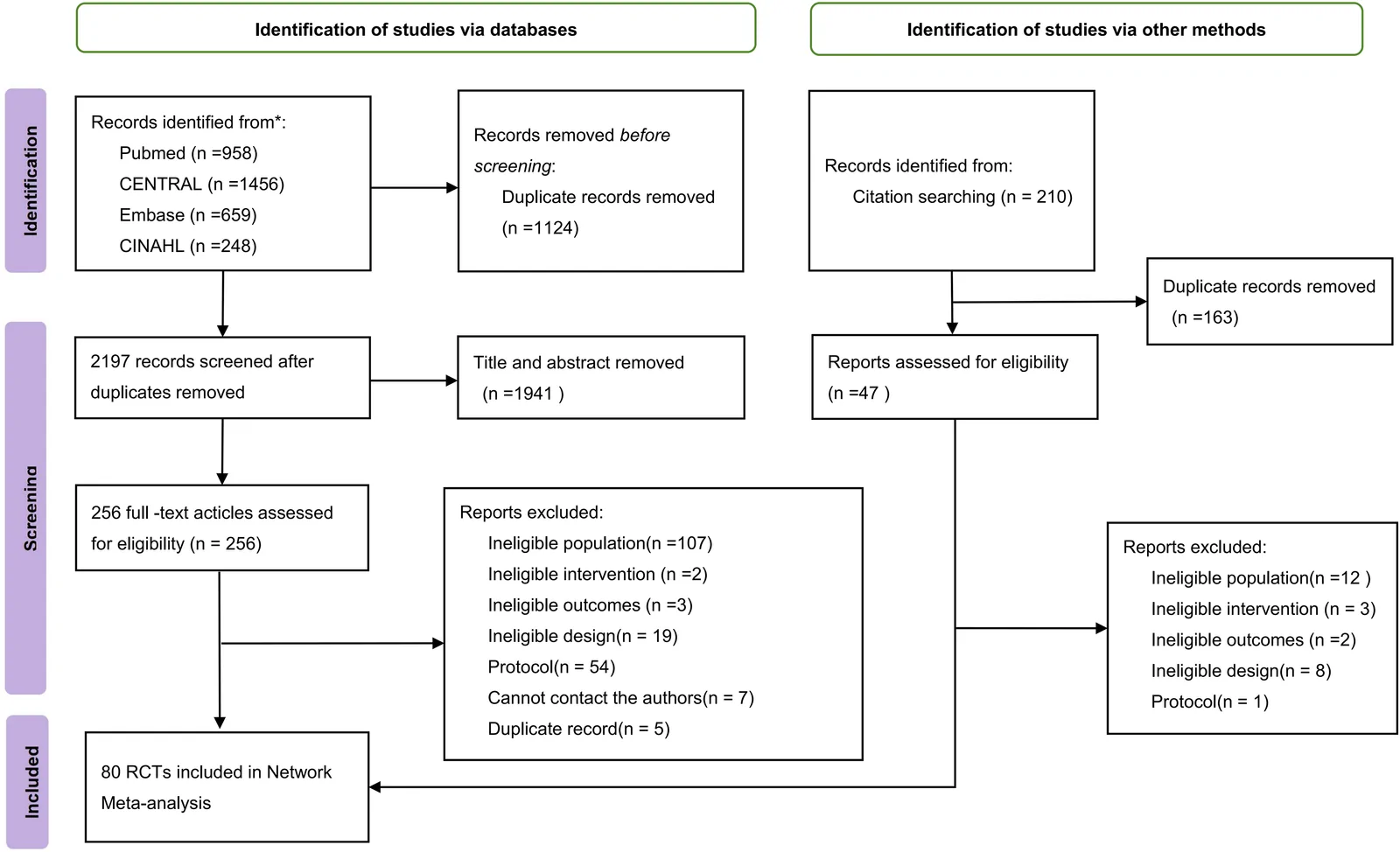
PRISMA diagram. This is the flowchart of the preliminary literature selection process for this study.

This study is designed as a network meta-analysis to synthesize and compare available evidence on non-pharmacological interventions for CUD. To date, only preliminary literature searches and search strategy validations have been completed(Fig 1). Formal study selection, data extraction, and quantitative synthesis, including network meta-analysis and GRADE evidence assessments, have not yet commenced. All analytical work is scheduled for completion by September 2026, with manuscript preparation and submission to follow. Full study results will be presented in the final manuscript upon completion of the study.

## Discussion

Current clinical guidelines typically recommend psychosocial interventions as the primary non-pharmacological approach for CUD. However, existing frameworks offer limited granular guidance on specific protocols, such as CBT, MET, and CM, while largely overlooking the independent potential of neurotherapy in addiction recovery. This network meta-analysis aims to move beyond generic recommendations by establishing a head-to-head comparative hierarchy of non-pharmacological interventions for CUD. By integrating psychosocial therapies and neurobiological modalities into a unified network, our study will deliver precise effect-size estimates and probability rankings to inform personalized clinical decision-making.

As of this protocol submission, no preliminary empirical findings or quantitative syntheses are available, as formal study screening and data extraction have not yet commenced. Methodological rigor will be ensured through prospective protocol registration on PROSPERO, strict adherence to PRISMA-P guidelines, and standardized duplicate processes for study selection, data extraction, and quality appraisal. Methodological quality will be systematically assessed using the ROBUST-RCT tool, and the certainty of network evidence will be evaluated using the GRADE framework.

This study possesses several key methodological strengths. First, the comprehensiveness and rigor of our search strategy were cross-validated against a recently published benchmark systematic review evaluating CUD outcome domains [25]. Our retrieval strategy successfully captured over 90% of the psychological intervention trials included in that landmark review, demonstrating high sensitivity and accuracy. Second, we will employ the GRADE framework to evaluate the certainty of evidence and calculate absolute effect sizes to contextualize clinical relevance. Third, to prevent network fragmentation into disconnected single-study nodes while capturing implementation variations, trial-level operational features, such as treatment intensity, therapist contact, group versus individual settings, and digital delivery formats, alongside clinical modifiers like participant age and baseline CUD severity, will be systematically evaluated via network meta-regressions and sensitivity analyses rather than as node-defining criteria, thereby preserving statistical power while delivering actionable evidence for personalized care.

While this network meta-analysis employs a rigorous framework, several potential methodological pitfalls and their corresponding contingency plans should be acknowledged. First, high clinical heterogeneity may arise from variable neurotherapy parameters and diverse psychosocial protocols. To address this, we will conduct predefined network meta-regressions and subgroup analyses based on treatment duration, delivery format, and stimulation parameters. Second, potential network disconnection or sparse comparisons may occur due to a limited number of eligible trials. If the network is disconnected, pairwise meta-analyses will be performed for connected sub-networks, or nodes will be merged at a broader categorical level based on clinical consensus. Third, blinding is inherently difficult in psychosocial trials, which may introduce performance and detection bias. We will use the ROBUST-RCT instrument to assess risk of bias, applying predefined overall decision rules tailored to subjective and objective outcomes. The sensitivity analyses will be conducted by excluding trials with a high overall risk of bias. Fourth, inconsistent outcome measures and varying recall periods across trials may introduce measurement heterogeneity. To address this, all reported cannabis-use frequencies will be mathematically standardized to a uniform 30-day timeframe prior to synthesis, standardized mean differences will be applied for different continuous scales, and sensitivity analyses will exclude studies using non-standardized assessment tools.

## Conclusion

This network meta-analysis provides a unified, head-to-head comparative evaluation bridging psychosocial and neurobiological interventions for CUD. By pairing network estimates with rigorous GRADE certainty assessments, our study will identify optimal non-pharmacological strategies to guide tailored clinical decision-making. These insights will help standardize individual treatment protocols and inform high-priority targets for future trial research.

## Supporting information

S1 Table PRISMA-P 2015 Checklist. (PDF)

S2 Table Search strategies. (PDF)

S3 Table Data Extraction Form. (PDF)
