# Supplementary material for: Comparative efficacy of non-pharmacological interventions for individuals with cannabis use disorder: a systematic review and network meta-analysis protocol
Source: PLoS One. 2026 Aug 21;21(8):e0355941. doi: 10.1371/journal.pone.0355941 (PMC13496447; doi:10.1371/journal.pone.0355941)
Supplement: Supplementary file 2 [file pone.0355941.s002.pdf]

Text S1. Search strategies

Medline(PubMed)

	#	Searches	Results
**A.Cannabis use disorder**	1	(Marijuana Abuse[MeSH Terms]) OR (Cannabis Abuse[Title/Abstract] OR Cannabis Dependence[Title/Abstract] OR Hashish Abuse[Title/Abstract] OR Cannabis-Related Disorder[Title/Abstract])	7972
	2	(Marijuana Smoking[MeSH Terms]) OR (Marijuana Smoking[Title/Abstract] OR Cannabis Smoking[Title/Abstract] OR Hashish Smoking[Title/Abstract] OR Blunts Smoking[Title/Abstract])	6274
	3	(Cannabis[Title/Abstract] OR Marihuana[Title/Abstract] OR Marijuana[Title/Abstract] OR Hemp[Title/Abstract] OR Ganja[Title/Abstract] OR Bhang[Title/Abstract] OR Cannabis sativa[Title/Abstract] OR Cannabis indica[Title/Abstract] OR Hashish[Title/Abstract] OR cannabacae[Title/Abstract] OR charas[Title/Abstract]) AND (abstain*[Title/Abstract] OR abstinence[Title/Abstract] OR abuse*[Title/Abstract] OR addict*[Title/Abstract] OR detox*[Title/Abstract] OR disorder*[Title/Abstract] OR depend*[Title/Abstract] OR misuse*[Title/Abstract] OR overuse[Title/Abstract] OR withdrawal[Title/Abstract])	19290
	4	#1 OR #2 OR #3	26670
**B1.Non-pharmacological treatment（Psychological interventions）**	5	(cognitive behavioral therapy[MeSH Terms]) OR (Cognitive Behavioral Therap*[Title/Abstract] OR Cognitive?Therap*[Title/Abstract] OR Cognitive Psychotherap*[Title/Abstract] OR Cognitive Behaviour Therap*[Title/Abstract])	50819
	6	(Behavior Therapy[MeSH Terms]) OR (Behavior Therapie*[Title/Abstract] OR Behavior Change Technique*[Title/Abstract] OR Behavior Treatment[Title/Abstract] OR Conditioning Therap*[Title/Abstract] OR Behavior Modification*[Title/Abstract] OR Behavior-Change Intervention*[Title/Abstract] OR Behavioral Counseling[Title/Abstract] OR Behavioral Support[Title/Abstract] OR Behavioral Technique*[Title/Abstract])	103638
	7	(Mindfulness-Based Cognitive Therapy[MeSH Terms]) OR (Mindfulness-Based Cognitive Therapy[Title/Abstract])	4006
	8	(Psychotherapy[MeSH Terms]) OR (Psychotherap*[Title/Abstract])	251531
	9	(Mindfulness[MeSH Terms]) OR (Mindfulness Meditation*[Title/Abstract])	9103
	10	Counseling[MeSH Terms]	50921
	11	(Motivational Interviewing[MeSH Terms]) OR (Motivational Interviewing[Title/Abstract])	6692
	12	(Social support[MeSH Terms]) OR (Social Support*[Title/Abstract] OR Social Care[Title/Abstract] OR Online Social Support*[Title/Abstract] OR Perceived Social Support*[Title/Abstract])	130161
	13	(Spiritual practice*[Title/Abstract] OR Psychosocial Therapy[Title/Abstract] OR Non-pharmacological[Title/Abstract] OR Community Reinforcement[Title/Abstract] OR Contingency Management[Title/Abstract] OR Psychosocial Support[Title/Abstract] OR Psychosocial intervention*[Title/Abstract] OR Positive reinforcement strategie*[Title/Abstract] OR Computer delivery psychosocial intervention*[Title/Abstract] OR Extended counseling[Title/Abstract] OR Psychotherap*[Title/Abstract] OR psychosocial*[Title/Abstract] OR behaviour?therapy[Title/Abstract] OR reinforcement[Title/Abstract] OR motivation*[Title/Abstract] OR contingent*[Title/Abstract] OR biofeedback[Title/Abstract] OR education*[Title/Abstract] OR brief intervention[Title/Abstract] OR	1390779

		early intervention[Title/Abstract] OR minimal intervention[Title/Abstract] OR counsel*[Title/Abstract] OR cognitive therapy[Title/Abstract] OR family therapy[Title/Abstract] OR stress management training[Title/Abstract] OR supportive expressive therapy[Title/Abstract] OR neurobehavioral*[Title/Abstract] OR coping skill*[Title/Abstract] OR self-control training[Title/Abstract] OR Digital intervention*[Title/Abstract])	
	14	#5 OR #6 OR #7 OR #8 OR #9 OR #10 OR #11 OR #12 OR #13 OR #14	1651817
**B2.Non-pharmacological treatment （Alternative therapies）**	15	(Complementary Therapies[MeSH Terms]) OR (Complementary Medicine[Title/Abstract] OR Alternative Medicine[Title/Abstract] OR Complementary Therap*[Title/Abstract] OR Alternative Therap*[Title/Abstract])	287124
	16	(Acupuncture[MeSH Terms]) OR (Acupuncture Treatment*[Title/Abstract] OR Pharmacopuncture[Title/Abstract] OR Pharmacoacupuncture Treatment[Title/Abstract] OR Acupotom*[Title/Abstract] OR Auricular Acupuncture*[Title/Abstract] OR Acupuncture Point*[Title/Abstract] OR Acupoint*[Title/Abstract] OR Acupuncture Anesthesia[Title/Abstract])	35943
	17	Electroacupuncture[MeSH Terms]	5587
	18	(Moxibustion[MeSH Terms]) OR (Moxibustion[Title/Abstract]) OR (Moxabustion[Title/Abstract])	4426
	19	(Massage[MeSH Terms]) OR (Massage?Therap*[Title/Abstract] OR Zone Therap*[Title/Abstract])	8264
	20	(Cupping Therapy[MeSH Terms]) OR (Cupping Therap*[Title/Abstract] OR Cupping Treatment*[Title/Abstract] OR baguan[Title/Abstract])	449
	21	(Qigong[MeSH Terms]) OR (Qigong[Title/Abstract] OR Qi Gong[Title/Abstract] OR Ch'i Kung[Title/Abstract])	1395
	22	(Tai Ji[MeSH Terms]) OR (Tai Chi[Title/Abstract] OR Tai Ji Quan[Title/Abstract] OR Taijiquan[Title/Abstract] OR T'ai Chi[Title/Abstract] OR Tai Chi Chuan[Title/Abstract])	3047
	23	(Deep Brain Stimulation[MeSH Terms]) OR (Deep Brain Stimulation*[Title/Abstract] OR Electrical Stimulation Brain[Title/Abstract])	19215
	24	Transcranial Magnetic Stimulation[MeSH Terms] OR Transcranial Magnetic Stimulation*[Title/Abstract]	24683
	25	(Transcranial Direct Current Stimulation[MeSH Terms]) OR (tDCS[Title/Abstract] OR Anodal Stimulation tDCS[Title/Abstract] OR Cathodal Stimulation tDCS[Title/Abstract] OR Transcranial Alternating Current Stimulation[Title/Abstract] OR Transcranial Random Noise Stimulation[Title/Abstract] OR Repetitive Transcranial Electrical Stimulation[Title/Abstract] OR Transcranial Electrical Stimulation[Title/Abstract])	10709
	26	(Meditation[MeSH Terms]) OR (Transcendental Meditation[Title/Abstract])	4442
	27	Baduanjin[Title/Abstract] OR tuina[Title/Abstract] OR turina[Title/Abstract]	807
	28	#17 OR #18 OR #19 OR #20 OR #21 OR #22 OR #23 OR #24 OR #25 OR #26 OR #27 OR #28 OR #29	343667
**B3.Non-pharmacological treatment （Physical therapies）**	29	(Exercise[MeSH Terms]) OR (Physical Exercise*[Title/Abstract] OR Aerobic Exercise*[Title/Abstract] OR Isometric Exercise*[Title/Abstract] OR Acute Exercise*[Title/Abstract] OR Exercise Training[Title/Abstract] OR Physical Activity[Title/Abstract])	398665
	30	(Sport[MeSH Terms]) OR Athletic*[Title/Abstract])	246537
	31	#29 OR #30	515392
**B4.Non-pharmacological treatment （Lifestyle adjustments）**	32	(Diet Therapy[MeSH Terms]) OR (Diet Therap*[Title/Abstract] OR Dietary Modification*[Title/Abstract] OR Diet Therapy Restrictive[Title/Abstract] OR Dietary Restriction[Title/Abstract])	78386
	33	Sleep intervention*[Title/Abstract]	980
	34	#32 OR #33	76364
**B1+B2+B3+B4**	35	#14 OR #28 OR #31 OR #34	2429526

**C. RCT filter**	36	(Randomized Controlled Trial[MeSH Terms] OR Clinical Trial[MeSH Terms] OR Equivalence Trial[MeSH Terms] OR Pragmatic Clinical Trial[MeSH Terms] OR RCT[Title/Abstract] OR Random*[Title/Abstract] OR Placebo[Title/Abstract])	1265709
**RCT-Results (A+B+C)**	37	#4 AND #35 AND #36	958

Cochrane Central Register of Controlled Trials(Including WHO International Clinical Trial Registry)

	No.	Query	Results
**A. Cannabis use disorder**	1	[mh "Marijuana Abuse"]	891
	2	("Cannabis Abuse" OR "Cannabis Dependence" OR "Hashish Abuse" OR "Cannabis-Related Disorder"):ti,ab,kw	243
	3	[mh "Marijuana Smoking"]	466
	4	("Marijuana Smoking" OR "Cannabis Smoking" OR "Hashish Smoking" OR "Blunts Smoking"):ti,ab,kw	504
	5	(Cannabis OR Marihuana OR Marijuana OR Hemp OR Ganja OR Bhang OR "Cannabis sativa" OR "Cannabis indica" OR Hashish OR cannabacae OR charas):ti,ab,kw	4874
	6	(abstain* OR abstinence OR abuse* OR addict* OR detox* OR disorder* OR depend* OR misuse* OR overuse OR withdrawal):ti,ab,kw	377935
	7	#5 AND #6	3342
	8	#1 OR #2 OR #3 OR #4 OR #7	3541
**B1.Non-pharmacological treatment （Psychological interventions）**	9	[mh "cognitive behavioral therapy"]	22398
	10	("Cognitive Behavioral Therapy" OR "Cognitive Therapy" OR "Cognitive Psychotherapy" OR "Cognitive Behavior Therapy"):ti,ab,kw	26476
	11	[mh "Behavior Therapy"]	14820
	12	("Behavior Therapy" OR "Behavior Change Technique" OR "Behavior Treatment" OR "Conditioning Therapy" OR "Behavior Modification" OR "Behavior-Change Intervention" OR "Behavioral Counseling" OR "Behavioral Support" OR "Behavioral Technique"):ti,ab,kw	15864
	13	[mh "Mindfulness-Based Cognitive Therapy"]	1072
	14	(Mindfulness-Based Cognitive Therapy):ti,ab,kw	1001
	15	Psychotherapy	20707
	16	(Psychotherap*):ti,ab,kw	18439
	17	Mindfulness	11134
	18	("Mindfulness Meditation"):ti,ab,kw	1313
	19	Counseling	32073
	20	"Motivational Interviewing"	5586
	21	("Motivational Interviewing"):ti,ab,kw	5313
	22	"Social support"	11922
	23	("Social Support" OR "Social Care" OR "Online Social Support" OR "Perceived Social Support"):ti,ab,kw	12547
	24	("Spiritual practice" OR "Psychosocial Therapy" OR "Non-pharmacological" OR "Community Reinforcement" OR "Contingency Management" OR "Psychosocial Support" OR "Psychosocial intervention" OR "Positive reinforcement strategie" OR "Computer delivery psychosocial	217951

		intervention" OR "Extended counseling" OR "Psychotherapy" OR "psychosocial" OR "behavior therapy" OR reinforcement OR motivation* OR contingent* OR biofeedback OR education* OR "brief intervention" OR "early intervention" OR "minimal intervention" OR counsel* OR "cognitive therapy" OR "family therapy" OR "stress management training" OR "supportive expressive therapy" OR neurobehavioral* OR "coping skill" OR "self-control training" OR "Digital intervention"):ti,ab,kw	
	25	#12 OR #13 OR #14 OR #15 OR #16 OR #17 OR #18 OR #19 OR #20 OR #21 OR #22 OR #23 OR #24 OR #25	242972
**B2.Non-pharmaco logical treatment （Alternative therapies）**	26	"Complementary Therapies"	2940
	27	("Complementary Medicine" OR "Alternative Medicine" OR "Complementary Therapy" OR "Alternative Therapy"):ti,ab,kw	4806
	28	Acupuncture OR Electroacupuncture OR Moxibustion OR Massage OR "Cupping Therapy" OR Qigong OR Tai Ji OR "Deep Brain Stimulation" OR "Transcranial Magnetic Stimulation" OR "Transcranial Direct Current Stimulation" OR Meditation	56224
	29	("Acupuncture Treatment" OR Pharmacupuncture OR "Pharmacupuncture Treatment" OR Acupotom* OR "Auricular Acupuncture" OR "Acupuncture Point" OR Acupoint* OR "Acupuncture Anesthesia"):ti,ab,kw	10247
	30	(Moxibustion OR Moxabustion):ti,ab,kw	2759
	31	(Massage?Therap* OR "Zone Therapy" OR "Cupping Therapy" OR "Cupping Treatment" OR baguan OR Qigong OR "Qi Gong" OR "Ch'i Kung" OR "Tai Chi" OR "Tai Ji Quan" OR Taijiquan OR "T'ai Chi" OR "Tai Chi Chuan"):ti,ab,kw	3279
	32	("Deep Brain Stimulation" OR "Electrical Stimulation Brain" OR "Transcranial Magnetic Stimulation" OR tDCS OR "Anodal Stimulation tDCS" OR "Cathodal Stimulation tDCS" OR "Transcranial Alternating Current Stimulation" OR "Transcranial Random Noise Stimulation" OR "Repetitive Transcranial Electrical Stimulation" OR "Transcranial Electrical Stimulation" OR "Transcendental Meditation"):ti,ab,kw	16398
	33	(Baduanjin OR tuina OR turina):ti,ab,kw	818
	34	#27 OR #28 OR #29 OR #30 OR #31 OR #32 OR #33 OR #34	65812
**B3.Non-pharmaco logical treatment （Physical therapies）**	35	Exercise OR Sport	153110
	36	("Physical Exercise" OR "Aerobic Exercise" OR "Isometric Exercise" OR "Acute Exercise" OR "Exercise Training" OR "Physical Activity" OR Athletic):ti,ab,kw	76159
	37	#36 OR #37	177277
**B4.Non-pharmaco logical treatment （Lifestyle adjustments）**	38	"Diet Therapy"	14360
	39	("Diet Therapy" OR "Dietary Modification" OR "Diet Therapy Restrictive" OR "Dietary Restriction"):ti,ab,kw	15070
	40	("Sleep intervention"):ti,ab,kw	519
	41	#39 OR #40 OR #41	15700
**B1+B2+B3+B4**	42	#26 OR #35 OR #38 OR #42	440653
**A+B**	43	#8 AND #42	1631
**C. filter**	44	Conference proceeding:pt or abstract:so	258601
**D. Results**	45	(#43 not #44) in Cochrane Reviews and Trials	1459

Embase.com

	No.	Query	Results
**A:Cannabis use disorder**	#1	'Marijuana Abuse'/exp	13038
	#2	'Marijuana Smoking'/exp	4561
	#3	('Mari？uana Abuse' OR 'Cannabis Abuse' OR 'Cannabis Dependence' OR 'Marijuana Dependence' OR 'Hashish Abuse' OR 'Cannabis Related Disorder'):ti,ab,kw	2365
	#4	('Marihuana Smoking' OR 'Cannabis Smoking' OR 'Hashish Smoking' OR 'Blunt* Smoking*'):ti,ab,kw	692
	#5	(Cannabis OR Hemp Plant* OR Bhang* OR Cannabis sativa OR Cannabis indica OR Hashish* OR cannabacae OR ganja* OR charas):ti,ab,kw AND (abstain* or abstinen* or abuse* or addict* or detox* or disorder* or depend* or habitual* or misuse* or overuse or withdrawal):ti,ab,kw	708
	#6	#1 OR #2 OR #3 OR #4 OR #5	18257
**B1.Non-pharmacological treatment （Psychological interventions）**	#7	'cognitive behavioral therapy'/exp	40415
	#8	("CBT" OR "cognitive behavior" OR "cognitive behaviour"):ti,ab,kw	34191
	#9	('Behavior Therapy' OR 'Mindfulness-Based Cognitive Therapy' OR 'Psychotherapy' OR 'Psychosocial intervention' OR 'Mindfulness' OR 'Mindfulness-based intervention' OR 'Counselling' OR 'Motivational Interviewing' OR 'Social support')/exp	683995
	#10	(('behavior' OR 'behaviour') AND ('therapy' OR 'treatment' OR 'psychotherapy')) :ti,ab,kw	260375
	#11	'mindfulness' AND ('CBT' OR 'cognitive therapy' OR 'cognitive behavior therapy' OR 'cognitive behaviour therapy' OR "intervention" OR "therapy"):ti,ab,kw	14899
	#12	('holistic psychotherapy' OR 'multiple psychotherapy' OR 'psychotherapeutic processes' OR 'psychotherapeutic training' OR 'socioenvironmental therapy'):ti,ab,kw	336
	#13	(('psycho-social' OR 'psychosocial') AND ('intervention' OR 'therapy' OR 'treatment')):ti,ab,kw	90667
	#14	('counselling' OR 'counseling' OR 'motivational interviewing' OR 'social support'):ti,ab,kw	282669
	#15	('spiritual practices' OR 'non-pharmacological' OR 'community reinforcement' OR 'contingency management' OR 'psychosocial support' OR 'positive reinforcement strategie*' OR 'brief intervention' OR 'family therapy' OR 'stress management training' OR 'supportive expressive therapy' OR 'neurobehavioral*' OR 'self-control training' OR 'digital intervention*'):ti,ab,kw	75153
	#16	#7 or #8 or #9 or #10 or #11 or #12 or #13 or #14 or #15	1106053
**B2.Non-pharmacological treatment (Alternative therapies）**	#17	('Complementary Therapies' OR 'Acupuncture' OR 'Electroacupuncture' OR 'Warm acupuncture' OR 'Auricular acupuncture' OR 'Tui Na' OR 'Moxibustion' OR 'Massage' OR 'Cupping Therapy' OR 'Qigong' OR 'Tai Ji' OR 'Brain depth stimulation' OR 'Transcranial Magnetic Stimulation' OR 'Ranscranial Direct Current Stimulation' OR 'Transcranial electrical stimulation' OR 'Meditation')/exp	272290
	#18	('alternative therap*' OR 'complementary medicine' OR 'complementary therap*' OR 'eclectic medicine' OR 'historical eclecticism' OR 'mental healing' OR 'mind	61762

		body technique' OR 'mind body therap*' OR 'mind-body relations ' OR 'polarity therapy' OR 'radi?esthesia' OR 'therapeutic cults' OR 'thomsonian medicine' OR 'alternative medicine'):ti,ab,kw	
	#19	('needle therapy' OR 'fire acupuncture' OR 'fire needling' OR 'heat?acupuncture' OR 'therm?acupuncture' OR 'needle acupuncture' OR 'warm acupuncture'):ti,ab,kw	955
	#20	('earlobe acupuncture' OR 'auriculo?acupuncture' OR 'auriculotherapy' OR 'ear acupuncture'):ti,ab,kw	855
	#21	('massage' OR 'cupping'):ti,ab,kw	22569
	#22	('chi kung' OR 'chigung' OR 'qigong' OR 'Tai Ji' OR 'Tai Chi'):ti,ab,kw	5289
	#23	('brain excitation' OR 'brain stimul*' OR 'brain stimulation' OR 'brain stimulation' OR 'transcranial magnetic stimulation' OR 'tDCS' OR 'transcranial direct current stimulation'):ti,ab,kw	78828
	#24	('Baduanjin' OR 'tuina'):ti,ab,kw	1059
	#25	#17 OR #18 OR #19 OR #20 OR #21 OR #22 OR #23 OR #24	321441
**B3.Non-pharmacological treatment(Physical therapies)**	#26	'exercise' OR 'sport'/exp	946816
	#27	('exertion' OR 'fitness training' OR 'fitness workout' OR 'physical effort' OR 'physical exertion' OR 'physical workout' OR 'exercise' OR 'sport'):ti,ab,kw	579126
	#28	#26 OR #27	978529
**B4.Non-pharmacological treatment(Lifestyle adjustments)**	#29	'Diet Therapy'/exp	481634
	#30	('diet* intervention*' OR 'diet* treatment' OR 'diet* therapy' OR 'nutrition* therapy'):ti,ab,kw	46368
	#31	#29 OR #30	499395
**B1+B2+B3+B4**	#32	#16OR #25 OR #28 OR #31	2716104
**A+B**	#33	#6 AND #32	2843
**C. RCT filter**	#34	'randomized controlled trial'/exp	997931
	#35	(Clinical Trial OR Equivalence Trial OR Pragmatic Clinical Trial OR RCT OR Random* OR Placebo):ti,ab,kw	2816833
	#36	#34 OR #35	2925945
**Results (A+B+C)**	#37	#33 AND #36	737
	#38	#37 NOT ([preprint]/lim OR 'conference abstract'/it OR 'conference paper'/it OR 'conference review'/it)	659

CINAHL

	No.	Query	Limiters/Expanders
**A:Cannabis use disorder**	#1	TI("Cannabis use disorder" or "Marijuana Abuse" or "Marijuana Smoking")	Search modes - Find all my search terms
	#2	(SU"cannabis" OR TI("mari?uana" OR "hemp plant*" OR "ganja*" OR "bhang*" OR "hemp*" OR "cannabacae" OR "hash*" OR "bhang" OR "charas")) AND (TI ("abstain*" OR "abstinen*" OR "abuse*" OR "addict*" OR "detox*" OR "disorder*" OR "depend*" OR "habitual*" OR "misuse*" OR "overuse or withdrawal"))	Search modes - Find all my search terms
	#3	#1 OR #2	Search modes - Find all my search terms
**B1.Non-pharmacological treatment(Psychological interventions)**	#4	SU("Cognitive Therapy" OR "Behavior Therapy")	Search modes - Find all my search terms
	#5	SU(Psychotherapy" OR "Psychosocial Intervention")	Search modes - Find all my search terms
	#6	SU("Mindfulness" OR "Mind Body Techniques" OR "Acceptance and Commitment Therapy")	Search modes - Find all my search terms
	#7	SU("Counseling")	Search modes - Find all my search terms
	#8	SU("Motivational Interviewing" OR "Craving")	Search modes - Find all my search terms
	#9	SU("Social Support " OR "Family Support" OR "Community Support" OR " Psychosocial Support")	Search modes - Find all my search terms
	#10	SU("Early Intervention" OR "Brief Psychotherapy" OR "Internet-Based Intervention")	Search modes - Find all my search terms
	#11	SU("Digital Health")	Search modes - Find all my search terms
	#12	TI("Spiritual practices" OR "Psychosocial Therapy" OR "Non-pharmacological" OR "Community Reinforcement" OR "Contingency Management" OR "Positive reinforcement strategy" OR "family therapy" OR "stress management training" OR "supportive expressive therapy" OR "coping skill" OR "self-control training" OR "Digital intervention" OR "mindfulness-based intervention")	Search modes - Find all my search terms
**B2.Non-pharmacological treatment (Alternative therapies）**	#13	SU("Alternative Therapies" OR "Massage Therapy" OR "Cupping Therapy")	Search modes - Find all my search terms
	#14	SU("Acupuncture" OR "Dry Needling" OR "Electroacupuncture" OR "Acupressure" OR "Auriculotherapy" OR "Acupuncture Points")	Search modes - Find all my search terms
	#15	SU("Tui Na" OR "Moxibustion" OR "Massage" OR "Physical Therapists" OR "Meditation" OR "Mindfulness" OR MH "Yoga" OR "Qigong" OR "Tai Chi")	Search modes - Find all my search terms
	#16	SU("Deep Brain Stimulation" OR "Electric* Stimulation" OR "Transcranial Magnetic Stimulation" OR"Transcutaneous Electric Nerve Stimulation" OR "tDCS" OR "transcranial direct current stimulation")	Search modes - Find all my search terms
	#17	TI("Complementary Therapies" OR "Baduanjin")	Search modes - Find all my search terms
**B3.Non-phar**	#18	SU("Physical Therapy" OR "Exercise")	Search modes - Find all my

**macological treatment(Physical therapies)**			search terms
**B4.Non-pharmacological treatment(Lifestyle adjustments)**	#19	SU("Diet Therapy")	Search modes - Find all my search terms
**B1+B2+B3+B4**	#20	#4 OR #5 OR #6 OR #7 OR #8 #9 OR #10 OR #11 OR #12 OR #13 OR #14 OR #15 OR #16 OR #17 OR #18 OR #19	Search modes - Find all my search terms
**C:RCT**	#21	SU("Clinical Trials+")	Search modes - Find all my search terms
	#22	SU("Random Assignment")	Search modes - Find all my search terms
	#23	SU("Placebos")	Search modes - Find all my search terms
	#24	TI("randomized controlled trial" or "controlled clinical trial" or "randomized" or "placebo" or "clinical trial" or "random*" or "trial")	Search modes - Find all my search terms
	#25	#21 OR #22 OR #23 OR #24	Search modes - Find all my search terms
**A+B+C**	#26	#3 AND #20 OR #25	Search modes - Find all my search terms
