# Supplementary material for: Comparative efficacy of non-pharmacological interventions for individuals with cannabis use disorder: a systematic review and network meta-analysis protocol
Source: PLoS One. 2026 Aug 21;21(8):e0355941. doi: 10.1371/journal.pone.0355941 (PMC13496447; doi:10.1371/journal.pone.0355941)
Supplement: Supplementary file 3 [file pone.0355941.s003.pdf]

Appendix Table S3

The Standardized Data Extraction Form of Non-pharmacological Interventions for cannabis use disorder

ID	First author/ Year	Register number	Funding	Country of Patients	Disease of Patients	Comorbidity	Age,mean (SD)	Gender,Male sex(%)	Sample Size	Interventions （dosages,frequencies,and delivery formats）	Study period	Outcomes	Adverse events	The definition of NPIs
