# Supplementary material for: Comparative efficacy of non-pharmacological interventions for individuals with cannabis use disorder: a systematic review and network meta-analysis protocol
Source: PLoS One. 2026 Aug 21;21(8):e0355941. doi: 10.1371/journal.pone.0355941 (PMC13496447; doi:10.1371/journal.pone.0355941)
Supplement: Supplementary file 4 [file pone.0355941.s005.docx]

Subject: Response to Reviewers – Manuscript ID [PONE-D-26-23451]

Dear Editor and Reviewers,

Thank you very much for your time and effort in reviewing our manuscript titled “[Comparative efficacy of non-pharmacological interventions for individuals with cannabis use disorder: a systematic review and network meta-analysis protocol]” (Manuscript ID: [PONE-D-26-23451]). We sincerely appreciate the insightful and constructive comments provided by the reviewers. We have carefully revised the manuscript accordingly and believe that the changes have substantially improved the quality and clarity of the work.

Below, we provide a point-by-point response to all comments. All changes have been highlighted in the revised manuscript.

**Response to Reviewer #1**

Comment 1: The manuscript refers to an “8-tier methodological framework.” Please provide a concise description or schematic to ensure readers understand how interventions will be categorized and compared.
**Response A1:** We sincerely thank reviewer for these comments regarding intervention taxonomy. Given overarching categories (such as a generic "8-tier framework" ) risk grouping clinically heterogeneous interventions and compromising the clinical granularity of the network, we have completely overhauled our classification framework. We removed the former 8-tier macro-level framework and replaced it with a highly refined, transparent intervention taxonomy. Modify as follows:

【Methods-Data extraction】“*We will extract the core components of each intervention and define them as network nodes, which will be further classified into first-level and second-level nodes. First-level nodes will represent commonly used single interventions as well as frequently applied two- or three-component combinations, thereby preserving network connectivity while avoiding excessive sparsity. These nodes encompassed specific behavioral, motivational, structural, neuromodulatory, and multi-component strategies (e.g., CBT, MET, CM, rTMS, transcranial direct current stimulation, or CBT+MET). Second-level nodes will provide a more granular representation of interventions, enabling sensitivity or subgroup analyses across different outcomes.Where applicable, second-level nodes will be prespecified for specific modalities (e.g., distinguishing abstinence-based CM from attendance-based CM).*

*Operational variations, including treatment intensity, duration, therapist contact, group versus individual setting, and face-to-face versus digital delivery, were systematically recorded. To prevent network disintegration into disconnected single-study nodes, these trial-level operational features were evaluated as potential sources of heterogeneity using pre-specified network meta-regressions and sensitivity analyses rather than serving as node-defining criteria.*”

(Page 11-12)

Comment 2: While primary outcomes are well defined, secondary outcomes include diverse measures (QoL, withdrawal, adverse events). Consider clarifying how heterogeneity across scales will be handled beyond standardization, particularly for QoL instruments.
**Response A2:** We sincerely thank reviewer for these comments regarding secondary outcomes.

First, we would like to clarify a conceptual ambiguity in our original text: Quality of Life (QoL) is not included as a primary or independent construct in this protocol. Instead, we evaluate cannabis-related problems using validated instruments such as the Cannabis-related Problems Scale(CPS) which captures functional impairments and psychosocial consequences. We have updated the protocol text to explicitly refer to "cannabis-related problems" rather than QoL to avoid conceptual confusion**(Page 8)**.

Second, to address heterogeneity across disparate measurement scales for secondary outcomes (Cannabis-related Problems Scale, withdrawal, adverse events), the specific revisions are as follows：

【Methods-Data synthesis and statistical analysis】“*To accommodate potential variations in scoring metrics and assessment tools across included trials, continuous secondary outcomes will be standardized prior to synthesis using Standardized Mean Differences (SMD) with 95% Confidence Intervals (CIs). Scale orientations will be systematically aligned so that higher scores will consistently represent greater symptom severity or functional impairment across all studies. Beyond metric standardization, measurement tools will be audited to ensure they measure clinically comparable constructs. Potential residual heterogeneity originating from scale variations will be systematically evaluated using random-effects models, network meta-regressions, and pre-specified sensitivity analyses omitting outlying assessment instruments.*

*For specific, narrative, or heterogeneously reported adverse events across primary studies, qualitative descriptive synthesis will be conducted. For standardized and consistently defined binary safety metrics, including the incidence of total adverse events, serious adverse events, or treatment discontinuation attributed to adverse events, quantitative network synthesis will be performed, with pooled effect estimates expressed as Risk Ratios (RRs) with 95% CIs.*”

**(Page 13-14)**.

Comment 3: The rationale for excluding quasi-experimental, adaptive, and cross-over designs is reasonable, but a brief explanation of how this exclusion may affect comprehensiveness would strengthen transparency.
**Response A3:** Thank you for this helpful comment. The specific revisions are as follows：

【Methods-Eligibility criteria】“*The quasi-experimental, adaptive, and cross-over designs will be excluded to preserve internal validity and satisfy the transitivity assumption across the network. Specifically, cross-over trials carry substantial risks of persistent carryover effects in cognitive-behavioral and neuromodulatory interventions, while non-randomized designs introduce marked selection bias. Although these strict restrictions minimize methodological heterogeneity, we acknowledge that excluding pragmatic or exploratory study designs may slightly limit the overall comprehensiveness of the retrieved trial landscape. The potential scope and implications of these exclusions will be formally discussed in the final review.*”(Page 9)

Comment 4: The current statement indicates data will be available upon request. To align with PLOS ONE’s policy, please clarify whether the minimal dataset will be deposited in a public repository upon completion.
**Response A4:** We thank the reviewer for this comment and have revised the Data Availability Statement in the manuscript to ensure full compliance with PLOS ONE’s policy, as detailed below: 【DATA AVAILABILITY STATEMENT】“*All relevant data supporting the planned systematic review and network meta-analysis will be made publicly available upon completion of the study. The minimal dataset, including standardized data extraction sheets, summary outcome data, and analytic scripts, will be deposited in a public repository such as the Open Science Framework (OSF) with a persistent DOI. No primary individual patient data are generated for this protocol.*”**(Page 24)**

Comment 5: Minor grammatical and stylistic edits would improve readability (e.g., streamline long sentences in the introduction and methods).

This protocol is methodologically sound and addresses a critical gap in comparative evidence for CUD interventions. With minor clarifications and improvements in reporting, it will provide a strong foundation for the planned systematic review and network meta-analysis. I recommend minor revisions to enhance clarity and compliance with journal standards.

**Response A5:** We sincerely thank the reviewer for the encouraging summary and constructive feedback.

In accordance with your recommendation, we have thoroughly proofread and revised the manuscript. Specifically, we streamlined complex and lengthy sentences across the Introduction and Methods sections, corrected minor grammatical inconsistencies, and enhanced sentence transitions to improve overall readability and conciseness. All revisions have been carefully made to strictly align with journal formatting and reporting standards without altering the core methodological substance.

**Response to Reviewer #2**

Comment 1: The population definition is too broad for cannabis use disorder
The protocol claims to study individuals with CUD, but the eligibility criteria allow inclusion of participants who used cannabis in the past month and had at least one cannabis-related problem. That is not equivalent to a formal diagnosis of CUD, cannabis dependence, or cannabis abuse. This could introduce major clinical heterogeneity and weaken the applicability of the findings.
The authors should separate formally diagnosed CUD from broader “problematic cannabis use” populations, or at least plan subgroup or sensitivity analyses by diagnostic certainty.
**Response B1:** We sincerely thank the reviewer for this helpful comment. The specific revisions are as follows：

【Methods-Eligibility criteria】“*To account for clinical heterogeneity, participants will be classified into formally diagnosed CUD cases (DSM or ICD criteria) and screening-identified problematic cannabis users (e.g., recruited from detoxification or emergency settings, or reporting past-month use with baseline problems).The primary analysis will synthesize all eligible populations combined. Pre-specified subgroup and sensitivity analyses will evaluate whether diagnostic certainty alters treatment estimates. If significant heterogeneity or subgroup differences are identified, findings will be stratified and reported separately by diagnostic certainty. Otherwise, the overall pooled synthesis will serve as the primary output*.”

**(Page 7)**

Comment 2: Age criteria are unclear and potentially inconsistent
The table defines adults as age ≥16 years, and later the manuscript discusses adolescents and young adults. In many jurisdictions, 16-year-olds are adolescents, not adults. The authors also plan a sensitivity analysis excluding adolescent trials, but the main inclusion criteria already mix adolescents and adults.
The authors should clearly define age groups and decide whether the review is about adults, adolescents, or both. If both are included, age should be treated as a major effect modifier in the NMA.

**Response B2:** We sincerely thank the reviewer for this helpful comment. The specific revisions are as follows：

【Methods-Eligibility criteria】“*Age groups will be explicitly categorized as adolescents (aged 12–17 years) and adults (aged ≥18 years). To address potential clinical heterogeneity driven by age differences, age will be evaluated as a effect modifier across primary outcomes. Network meta-regressions incorporating trial-level mean age will be conducted to detect potential effect modification. Additionally, pre-specified subgroup analyses stratified by age category (adolescents vs. adults) and sensitivity analyses excluding adolescent trials will be performed to ensure the validity of the final network rankings.*”**(Page 7)**

Comment 3: Outcomes are not sufficiently well defined
The primary outcomes are confusing. The abstract lists efficacy, acceptability, and cannabis-use days as primary outcomes. The methods describe efficacy as both abstinence and days of cannabis use. The table lists point abstinence, cannabis-use days, and retention. Secondary outcomes then include cannabis-use frequency over the past 30 days, which overlaps with cannabis-use days.
The authors should define one clear hierarchy of outcomes. For example:
Primary benefit outcome: biochemically verified abstinence at end of treatment.
Primary harm/acceptability outcome: all-cause dropout or treatment discontinuation.
Secondary outcomes: days of cannabis use, cannabis-related problems, dependence severity, craving, withdrawal, mood symptoms, adverse events.

**Response B3:** We sincerely thank the reviewer for this helpful comment. The specific revisions are as follows：

【Methods-Eligibility criteria】

“*Primary outcomes will evaluate intervention benefit, acceptability, and safety.*

1) *Primary benefit outcomes will include: (a) biochemically verified cannabis abstinence at the end of treatment, and (b) total days of cannabis use over the past 30 days (self-reported).*
2) *Primary Acceptability Outcome: Treatment retention, defined as the proportion of randomized participants who complete at least one session during the final week of treatment, based on an intention-to-treat approach. Additionally, retention at follow-up timepoints will be calculated as the proportion of randomized participants completing follow-up assessments without dropout.*
3) *Primary Safety/Harm Outcome: Overall incidence of adverse events (and serious adverse events) attributed to the intervention.*

*Secondary outcomes will capture consumption intensity and broader clinical impacts, including:*

1) *Quantity of cannabis use: Measured as the number of joints or grams consumed over the past 30 days.*
2) *Cannabis-related problems: Assessed via validated instruments such as the Cannabis Problems Scale (CPS).*
3) *Withdrawal and psychopathology: Withdrawal severity and associated psychological symptoms (including dependence, anxiety, and depression)* ”

**(Page 8)**

Comment 4: Intervention node classification is underdeveloped
The eight intervention categories are potentially useful, but the node-building rules are not precise enough. For example, “combined interventions” may include CBT plus MET, CBT plus CM, MET plus CM, family therapy plus CM, or psychosocial intervention plus neuromodulation. These are clinically very different and may not be valid as one node.
The authors should provide explicit rules for classifying multicomponent interventions, including how they will handle intensity, duration, therapist contact, delivery mode, group versus individual format, digital versus face-to-face delivery, and co-interventions.

**Response B4:** We sincerely thank reviewer for these comments regarding intervention node classification. Given overarching categories (such as a generic "8-tier framework" ) risk grouping clinically heterogeneous interventions and compromising the clinical granularity of the network, we have completely overhauled our classification framework. We removed the former 8-tier macro-level framework and replaced it with a highly refined, transparent intervention taxonomy. Modify as follows:

【Methods-Data extraction】“*We will extract the core components of each intervention and define them as network nodes, which will be further classified into first-level and second-level nodes. First-level nodes will represent commonly used single interventions as well as frequently applied two- or three-component combinations, thereby preserving network connectivity while avoiding excessive sparsity. These nodes encompassed specific behavioral, motivational, structural, neuromodulatory, and multi-component strategies (e.g., CBT, MET, CM, rTMS, transcranial direct current stimulation, CBT+MET, or CBT+CM). Second-level nodes will provide a more granular representation of interventions, enabling sensitivity or subgroup analyses across different outcomes.Where applicable, second-level nodes will be prespecified for specific modalities (e.g., distinguishing abstinence-based CM from attendance-based CM).*

*Operational variations, including treatment intensity, duration, therapist contact, group versus individual setting, and face-to-face versus digital delivery, were systematically recorded. To prevent network disintegration into disconnected single-study nodes, these trial-level operational features were evaluated as potential sources of heterogeneity using pre-specified network meta-regressions and sensitivity analyses rather than serving as node-defining criteria*.”

**(Page 11-12)**

Comment 5: Statistical methods contain internal inconsistencies
The statistical section needs substantial revision. The authors describe a frequentist NMA but refer to “credible intervals,” which are usually Bayesian. They also mention weighted least squares with REML and then say they will apply the DerSimonian-Laird random-effects model. REML and DerSimonian-Laird are different estimators and should not be presented as the same approach.

**Response B5:** We sincerely thank the reviewer for identifying these statistical inconsistencies. We have fully revised the Statistical Analysis section to ensure internal consistency within a frequentist framework:

【Methods-Data synthesis and statistical analysis】“*Treatment effects will be synthesized using a random-effects network meta-analysis model, with between-study variance estimated via Restricted Maximum Likelihood (REML).*” **(Page 13)**

Comment 6: GRADE and imprecision rules require clarification
The GRADE plan is ambitious but not always clear. The manuscript mentions minimally clinically important differences, decision thresholds, and absolute effects, but the actual thresholds are not defined. The authors should specify the MID or decision threshold for each outcome before analysis.
The statement that outcomes informed by a single trial with a large statistically significant effect will be considered “high risk of bias due to potential small-sample effects” is conceptually unclear. Small-study effects relate more directly to imprecision, publication bias, or small-study bias, not necessarily risk of bias.

**Response B6:** We thank the reviewer for these insightful and critical methodological comments regarding our GRADE framework. The specific revisions are as follows：

1. **Removal of MIDs to Avoid Threshold Confusion:** To eliminate redundancy and potential threshold conflict within our minimally contextualized framework, we have removed references to minimally clinically important differences (MIDs).
2. **Explicit Decision Thresholds in Minimal Contextualization:** Within the minimally contextualized GRADE approach, explicit clinical decision thresholds will be established prior to analysis.Superiority and imprecision will be evaluated using pre-defined relative effect boundaries( RR ≥ 1.25 or ≤ 0.80; SMD ≥ 0.20 or ≤ -0.20) combined with evidence certainty, rather than relying solely on 95% confidence intervals excluding the null value.**（see pages 15 for details)**
3. **Definition and Thresholds for Absolute Effects:**“*Absolute effect estimates will be calculated for dichotomous outcomes by applying pooled network relative estimates (RR) to the baseline risk of the control group, expressed as absolute risk differences per 1,000 participants over the treatment period. To evaluate imprecision for absolute outcomes, an absolute difference boundary of 50 events per 1,000 participants (5%) will serve as the decision threshold for clinical importance.*” **(Page 14)**
4. **Conceptual Correction for Small-Sample Effects:** We have corrected the misclassification regarding single small trials with large effect sizes.“*Outcomes informed by a single small trial with a large effect size will not be classified as high risk of bias; instead, they will be downgraded for imprecision or small-study bias during GRADE evaluation*.”**(Page 16）**

**Response to Reviewer #3**

Comment 1: The introduction of the study was carefully written, using relevant epidemiological data to situate the problem statement. Currently used interventions, mainly non-pharmacological treatments, were also presented. Nevertheless, although the work focuses on non-pharmacological treatment, current pharmacological treatments may be presented and evaluated, as in the non-pharmacological studies. Additionally, the authors may present some merits and demerits of the pharmacological treatments, which may skewed the study towards non-pharmacological interventions in the first instance.
**Response C1:** We sincerely thank the reviewer for this helpful comment. The specific revisions are as follows：

【Introduction】“*In parallel, pharmacological options like synthetic cannabinoids and gabapentin have been explored, offering potential merits in mitigating acute withdrawal symptoms and craving; however, their clinical adoption remains constrained by key demerits, including modest overall efficacy, adverse effect risks, and a lack of formal regulatory approval. Consequently, NPIs remain the frontline standard of care, though they are not universally curative, often require repeated sessions, and carry substantial resource demands.*”**(Page 4）**

Comment 2: The methodology is rigorous and dense, providing all relevant information to ensure reproducibility. The lack of language restrictions in the search strategies may raise questions, particularly with conclusions, as specific meanings may be lost in translations. Authors should justify why there were no restrictions on language. Also, on line 105, “from inception to 30 December, 2025”, the study period appears to have passed, or the authors may have made a typographical error.
**Response C2:** We sincerely thank the reviewer for this helpful comment. The specific revisions are as follows：

1. **Justification for Removing Language Restrictions:** Language restrictions were omitted to ensure comprehensive evidence retrieval and minimize potential language bias. While minor nuances could theoretically be affected during translation, full-text articles of non-English studies will be translated using validated translation procedures and verified by native or fluent researchers where necessary. Potential translation-related limitations will be explicitly addressed in the discussion section of the final manuscript.
2. **Clarification on the Search Date Range:** The date "30 December, 2025" reflects the cutoff date for our preliminary search, which was conducted during protocol development to verify search sensitivity and study yield. Prior to performing the final meta-analysis, the search will be fully updated to ensure all eligible studies up to the final analysis date are included.

【Methods-Search strategy】“*We preliminarily searched PubMed, Embase, the Cochrane Central Register of Controlled Trials (CENTRAL), and the Cumulative Index to Nursing and Allied Health Literature (CINAHL) from inception to December 30, 2025, without language restrictions. Prior to the final meta-analysis, the search will be fully updated to capture all newly published eligible studies.*” **(Page 6)**

Comment 3: The authors appear to intermix the future tense (will) and the past tense, resulting in some confusion as to whether the work has already been carried out or is a protocol to be carried out.
**Response C3:** We appreciate the reviewer pointing out this inconsistency. We clarify that this manuscript represents a study protocol. At the present stage, we have only conducted preliminary database searches and search strategy validation; no formal study selection, data extraction, or statistical synthesis has been carried out. To eliminate any confusion, we have thoroughly audited the tense usage throughout the manuscript, particularly in the Methods section.

Comment 4: A sample of the data extraction form (line 185) should be included.

**Response C4:** We sincerely thank the reviewer for this helpful comment. We have added an explicit reference to the data extraction template in the Methods section and included the complete sample form in the Supplementary Material as Table S1 (Sample Data Extraction Form).

【Methods-Data extraction】“*Two investigators will independently extract data in duplicate using a standardized form (Text S2), with disagreements resolved by consensus. We will extract study and participant characteristics, intervention details, and patient-reported outcomes. For continuous data, adjusted least-squares or baseline-adjusted end-scores will be prioritized, followed by change-from-baseline scores, and unadjusted end-scores.* ”**(Page 11)**

Comment 5: Lines 194 to 196: Please include a reference or justify why the control groups are split evenly. What are the implications if the groups are uneven?

**Response C5:** We thank the reviewer for this insightful comment. Splitting the shared control group is a standard method recommended by the Cochrane Handbook to prevent unit-of-analysis errors and the double-counting of control participants.We have updated methods and added the reference accordingly.

【Methods-Data extraction】“*For multi-arm trials with a shared control, the control group sample size will be split proportionally across intervention comparisons to prevent double-counting participants[17].*”**(Page 7)**

*[17] Higgins JPT, Thomas J, Chandler J, Cumpston M, Li T, Page MJ, et al., editors. Cochrane Handbook for Systematic Reviews of Interventions. Version 6.5 (updated January 2024). Cochrane; 2024. Available from: https://training.cochrane.org/handbook.*

**Response to Reviewer #4**

Comment 1: First, some acronyms are not defined at their first mention. For example, rTMS and tDCS should be fully written out when they first appear in the manuscript, followed by the abbreviation in parentheses.
**Response D1:** We thank the reviewer for pointing this out. We have thoroughly audited the manuscript to ensure all acronyms are fully defined at their first mention. Specifically, repetitive transcranial magnetic stimulation (rTMS) and transcranial direct current stimulation (tDCS) have now been spelled out in full upon their first appearance in the Introduction section, with their abbreviations following in parentheses.

Comment 2: Secondly, although this is a protocol, the manuscript would be strengthened if the authors could include any preliminary findings, pilot observations, or feasibility data, where available. If preliminary results are not yet available, the authors should explicitly state this and explain how the protocol will ensure methodological rigor.

**Response D2:**

We thank the reviewer for this helpful suggestion.

As this manuscript presents a prospective systematic review and network meta-analysis protocol, no preliminary empirical findings or outcome syntheses are currently available. To address the reviewer's comment, we have added an explicit statement in the revised manuscript clarifying that formal study selection and quantitative analyses have not yet commenced. The specific revisions are as follows：

【Discussion】“*As of this protocol submission, no preliminary empirical findings or quantitative syntheses are available, as formal study screening and data extraction have not yet commenced. Methodological rigor will be ensured through prospective protocol registration on PROSPERO, strict adherence to PRISMA-P guidelines, and standardized duplicate processes for study selection, data extraction, and quality appraisal. Methodological quality will be systematically assessed using the ROBUST-RCT tool, and the certainty of network evidence will be evaluated using the GRADE framework.*”**(Page 17)**

Comment 3: Lastly, the authors should briefly discuss possible methodological pitfalls and alternative strategies. For example, they may consider how they would address limited numbers of eligible randomized controlled trials, high heterogeneity across interventions, inconsistent outcome measures, small sample sizes, or disconnected networks in the network meta-analysis. A contingency plan/ alternative approach would improve confidence in the feasibility and robustness of the proposed approach.

**Response D3:** We thank the reviewer for this helpful suggestion. The specific revisions are as follows：

【Discussion】“*While this network meta-analysis employs a rigorous framework, several potential methodological pitfalls and their corresponding contingency plans should be acknowledged. First, high clinical heterogeneity may arise from variable neurotherapy parameters and diverse psychosocial protocols. To address this, we will conduct predefined network meta-regressions and subgroup analyses based on treatment duration, delivery format, and stimulation parameters. Second, potential network disconnection or sparse comparisons may occur due to a limited number of eligible trials. If the network is disconnected, pairwise meta-analyses will be performed for connected sub-networks, or nodes will be merged at a broader categorical level based on clinical consensus. Third, blinding is inherently difficult in psychosocial trials, which may introduce performance and detection bias. We will use the ROBUST-RCT instrument to assess risk of bias, applying predefined overall decision rules tailored to subjective and objective outcomes. The sensitivity analyses will be conducted by excluding trials with a high overall risk of bias.* ”**(Page 18)**

Once again, we thank the editor and reviewers for their valuable comments and suggestions. We hope that the revised manuscript meets the requirements for publication in [*PLOS ONE*].

Best regards,

Liming Lu (陸麗明)

Professor | MD

Guangzhou University of Chinese Medicine

232 Waihuan Dong Road Guangzhou 510006, China

Tel: +86 13751779800

Email: [lulimingleon@126.com](https://mail.126.com/js6/lulimingleon@126.com)
