# Supplementary material for: Comparative efficacy of non-pharmacological interventions for individuals with cannabis use disorder: a systematic review and network meta-analysis protocol
Source: PLoS One. 2026 Aug 21;21(8):e0355941. doi: 10.1371/journal.pone.0355941 (PMC13496447; doi:10.1371/journal.pone.0355941)
Supplement: Peer review history

# Peer review: Decision Letter 0

**DOI:** 10.1371/journal.pone.0355941.r001

**Authors:** Alberto Sá Filho

9 Jul 2026

Dear Dr. Lu,

Thank you for submitting your manuscript to PLOS ONE. After careful consideration, we feel that it has merit but does not fully meet PLOS ONE’s publication criteria as it currently stands. Therefore, we invite you to submit a revised version of the manuscript that addresses the points raised during the review process.

==============================

Thank you for submitting your manuscript entitled **“** Comparative efficacy of non-pharmacological interventions for individuals with cannabis use disorder: a systematic review and network meta-analysis protocol**”**  to *PLOS ONE* .

Your manuscript has now been evaluated by three reviewers. Both reviewers identified issues that require careful attention before the manuscript can be further considered for publication. Therefore, I invite you to submit a revised version addressing all comments raised by the reviewers in a detailed and point-by-point response letter.

In your revision, please ensure that all methodological, reporting, and conceptual concerns are fully addressed, with corresponding changes clearly incorporated into the manuscript where appropriate. Please also indicate precisely where each modification was made.

We look forward to receiving your revised manuscript and response to the reviewers’ comments.

==============================

Please submit your revised manuscript by Aug 08 2026 11:59PM. If you will need more time than this to complete your revisions, please reply to this message or contact the journal office at plosone@plos.org. When you're ready to submit your revision, log on to https://www.editorialmanager.com/pone/ and select the 'Submissions Needing Revision' folder to locate your manuscript file.

- A letter that responds to each point raised by the academic editor and reviewer(s). You should upload this letter as a separate file labeled 'Response to Reviewers'.
- A marked-up copy of your manuscript that highlights changes made to the original version. You should upload this as a separate file labeled 'Revised Manuscript with Track Changes'.
- An unmarked version of your revised paper without tracked changes. You should upload this as a separate file labeled 'Manuscript'.

If you would like to make changes to your financial disclosure, please include your updated statement in your cover letter. Guidelines for resubmitting your figure files are available below the reviewer comments at the end of this letter.

If applicable, we recommend that you deposit your laboratory protocols in protocols.io to enhance the reproducibility of your results. Protocols.io assigns your protocol its own identifier (DOI) so that it can be cited independently in the future. For instructions see: https://journals.plos.org/plosone/s/submission-guidelines#loc-laboratory-protocols. Additionally, PLOS ONE offers an option for publishing peer-reviewed Lab Protocol articles, which describe protocols hosted on protocols.io. Read more information on sharing protocols at https://plos.org/protocols?utm_medium=editorial-email&utm_source=authorletters&utm_campaign=protocols.

As the corresponding author, your ORCID iD is verified in the submission system and will appear in the published article. PLOS supports the use of ORCID, and we encourage all coauthors to register for an ORCID iD and use it as well. Please encourage your coauthors to verify their ORCID iD within the submission system before final acceptance, as unverified ORCID iDs will not appear in the published article. *Only*  the individual author can complete the verification step; PLOS staff *cannot*  verify ORCID iDs on behalf of authors.

We look forward to receiving your revised manuscript.

Kind regards,

Alberto Souza Sá Filho, Ph.D

Academic Editor

PLOS One

Journal Requirements:

When submitting your revision, we need you to address these additional requirements.

1. Please ensure that your manuscript meets PLOS ONE's style requirements, including those for file naming. The PLOS ONE style templates can be found at

https://journals.plos.org/plosone/s/file?id=wjVg/PLOSOne_formatting_sample_main_body.pdf and https://journals.plos.org/plosone/s/file?id=ba62/PLOSOne_formatting_sample_title_authors_affiliations.pdf

2. In the online submission form, you indicated that the datasets generated and analysed during the current study are available from the corresponding author upon reasonable request.

All PLOS journals now require all data underlying the findings described in their manuscript to be freely available to other researchers, either 1. In a public repository, 2. Within the manuscript itself, or 3. Uploaded as supplementary information.

This policy applies to all data except where public deposition would breach compliance with the protocol approved by your research ethics board. If your data cannot be made publicly available for ethical or legal reasons (e.g., public availability would compromise patient privacy), please explain your reasons on resubmission and your exemption request will be escalated for approval.

3. We note that the grant information you provided in the ‘Funding Information’ and ‘Financial Disclosure’ sections do not match.

When you resubmit, please ensure that you provide the correct grant numbers for the awards you received for your study in the ‘Funding Information’ section.

4. Thank you for stating the following financial disclosure:

This research was supported by Peak-Shaping Project under Guangzhou University of Chinese Medicine's Action Plan for Double First-Class and High-Level Disciplinary Development (GZY2025ZJ18); Guangdong Provincial Higher Education Innovation Team Project(2025KCXTD011); Shenzhen Medical Research Fund(C2501027); and Research and Practice on Optimization Strategies for a Clinical Recommendation System for Non-Pharmacological Interventions in Substance Use Disorders Driven by Large Language Models(A3-0317-25-109-009).

Please state what role the funders took in the study. If the funders had no role, please state: "The funders had no role in study design, data collection and analysis, decision to publish, or preparation of the manuscript."

If this statement is not correct you must amend it as needed.

Please include this amended Role of Funder statement in your cover letter; we will change the online submission form on your behalf.

5. Thank you for stating the following in the Acknowledgments Section of your manuscript:

This research was supported by Peak-Shaping Project under Guangzhou University of Chinese Medicine's Action Plan for Double First-Class and High-Level Disciplinary Development (GZY2025ZJ18); Guangdong Provincial Higher Education Innovation Team Project(2025KCXTD011); and Shenzhen Medical Research Fund(C2501027)

We note that you have provided funding information that is not currently declared in your Funding Statement. However, funding information should not appear in the Acknowledgments section or other areas of your manuscript. We will only publish funding information present in the Funding Statement section of the online submission form.

Please remove any funding-related text from the manuscript and let us know how you would like to update your Funding Statement. Currently, your Funding Statement reads as follows:

This research was supported by Peak-Shaping Project under Guangzhou University of Chinese Medicine's Action Plan for Double First-Class and High-Level Disciplinary Development (GZY2025ZJ18); Guangdong Provincial Higher Education Innovation Team Project(2025KCXTD011); Shenzhen Medical Research Fund(C2501027); and Research and Practice on Optimization Strategies for a Clinical Recommendation System for Non-Pharmacological Interventions in Substance Use Disorders Driven by Large Language Models(A3-0317-25-109-009).

Please include your amended statements within your cover letter; we will change the online submission form on your behalf.

6. If the reviewer comments include a recommendation to cite specific previously published works, please review and evaluate these publications to determine whether they are relevant and should be cited. There is no requirement to cite these works unless the editor has indicated otherwise. \

Reviewers' comments:

Reviewer's Responses to Questions

**Comments to the Author**

1. Does the manuscript provide a valid rationale for the proposed study, with clearly identified and justified research questions?

Reviewer #1: Yes

Reviewer #2: Yes

Reviewer #3: Yes

Reviewer #4: Yes

**********

2. Is the protocol technically sound and planned in a manner that will lead to a meaningful outcome and allow testing the stated hypotheses?

Reviewer #1: Yes

Reviewer #2: Partly

Reviewer #3: Yes

Reviewer #4: Yes

**********

3. Is the methodology feasible and described in sufficient detail to allow the work to be replicable?

Reviewer #1: Yes

Reviewer #2: Yes

Reviewer #3: Yes

Reviewer #4: Yes

**********

4. Have the authors described where all data underlying the findings will be made available when the study is complete?

The PLOS Data policy

Reviewer #1: Yes

Reviewer #2: Yes

Reviewer #3: Yes

Reviewer #4: Yes

**********

5. Is the manuscript presented in an intelligible fashion and written in standard English?

Reviewer #1: Yes

Reviewer #2: Yes

Reviewer #3: Yes

Reviewer #4: Yes

**********

Please use the space provided to explain your answers to the questions above and, if applicable, provide comments about issues authors must address before this protocol can be accepted for publication. You may also include additional comments for the author, including concerns about research or publication ethics.

You may also provide optional suggestions and comments to authors that they might find helpful in planning their study.

Reviewer #1: 1- The manuscript refers to an “8-tier methodological framework.” Please provide a concise description or schematic to ensure readers understand how interventions will be categorized and compared.

2- While primary outcomes are well defined, secondary outcomes include diverse measures (QoL, withdrawal, adverse events). Consider clarifying how heterogeneity across scales will be handled beyond standardization, particularly for QoL instruments.

3- The rationale for excluding quasi-experimental, adaptive, and cross-over designs is reasonable, but a brief explanation of how this exclusion may affect comprehensiveness would strengthen transparency.

4- The current statement indicates data will be available upon request. To align with PLOS ONE’s policy, please clarify whether the minimal dataset will be deposited in a public repository upon completion.

5- Minor grammatical and stylistic edits would improve readability (e.g., streamline long sentences in the introduction and methods).

This protocol is methodologically sound and addresses a critical gap in comparative evidence for CUD interventions. With minor clarifications and improvements in reporting, it will provide a strong foundation for the planned systematic review and network meta-analysis. I recommend minor revisions to enhance clarity and compliance with journal standards.

Reviewer #2: 1. The population definition is too broad for cannabis use disorder

The protocol claims to study individuals with CUD, but the eligibility criteria allow inclusion of participants who used cannabis in the past month and had at least one cannabis-related problem. That is not equivalent to a formal diagnosis of CUD, cannabis dependence, or cannabis abuse. This could introduce major clinical heterogeneity and weaken the applicability of the findings.

The authors should separate formally diagnosed CUD from broader “problematic cannabis use” populations, or at least plan subgroup or sensitivity analyses by diagnostic certainty.

2. Age criteria are unclear and potentially inconsistent

The table defines adults as age ≥16 years, and later the manuscript discusses adolescents and young adults. In many jurisdictions, 16-year-olds are adolescents, not adults. The authors also plan a sensitivity analysis excluding adolescent trials, but the main inclusion criteria already mix adolescents and adults.

The authors should clearly define age groups and decide whether the review is about adults, adolescents, or both. If both are included, age should be treated as a major effect modifier in the NMA.

3. Outcomes are not sufficiently well defined

The primary outcomes are confusing. The abstract lists efficacy, acceptability, and cannabis-use days as primary outcomes. The methods describe efficacy as both abstinence and days of cannabis use. The table lists point abstinence, cannabis-use days, and retention. Secondary outcomes then include cannabis-use frequency over the past 30 days, which overlaps with cannabis-use days.

The authors should define one clear hierarchy of outcomes. For example:

Primary benefit outcome: biochemically verified abstinence at end of treatment.

Primary harm/acceptability outcome: all-cause dropout or treatment discontinuation.

Secondary outcomes: days of cannabis use, cannabis-related problems, dependence severity, craving, withdrawal, mood symptoms, adverse events.

4. Intervention node classification is underdeveloped

The eight intervention categories are potentially useful, but the node-building rules are not precise enough. For example, “combined interventions” may include CBT plus MET, CBT plus CM, MET plus CM, family therapy plus CM, or psychosocial intervention plus neuromodulation. These are clinically very different and may not be valid as one node.

The authors should provide explicit rules for classifying multicomponent interventions, including how they will handle intensity, duration, therapist contact, delivery mode, group versus individual format, digital versus face-to-face delivery, and co-interventions.

5. Statistical methods contain internal inconsistencies

The statistical section needs substantial revision. The authors describe a frequentist NMA but refer to “credible intervals,” which are usually Bayesian. They also mention weighted least squares with REML and then say they will apply the DerSimonian-Laird random-effects model. REML and DerSimonian-Laird are different estimators and should not be presented as the same approach.

6. GRADE and imprecision rules require clarification

The GRADE plan is ambitious but not always clear. The manuscript mentions minimally clinically important differences, decision thresholds, and absolute effects, but the actual thresholds are not defined. The authors should specify the MID or decision threshold for each outcome before analysis.

The statement that outcomes informed by a single trial with a large statistically significant effect will be considered “high risk of bias due to potential small-sample effects” is conceptually unclear. Small-study effects relate more directly to imprecision, publication bias, or small-study bias, not necessarily risk of bias.

Reviewer #3: The introduction of the study was carefully written, using relevant epidemiological data to situate the problem statement. Currently used interventions, mainly non-pharmacological treatments, were also presented. Nevertheless, although the work focuses on non-pharmacological treatment, current pharmacological treatments may be presented and evaluated, as in the non-pharmacological studies. Additionally, the authors may present some merits and demerits of the pharmacological treatments, which may skewed the study towards non-pharmacological interventions in the first instance.

The methodology is rigorous and dense, providing all relevant information to ensure reproducibility. The lack of language restrictions in the search strategies may raise questions, particularly with conclusions, as specific meanings may be lost in translations. Authors should justify why there were no restrictions on language. Also, on line 105, “from inception to 30 December, 2025”, the study period appears to have passed, or the authors may have made a typographical error.

The authors appear to intermix the future tense (will) and the past tense, resulting in some confusion as to whether the work has already been carried out or is a protocol to be carried out.

A sample of the data extraction form (line 185) should be included.

Lines 194 to 196: Please include a reference or justify why the control groups are split evenly. What are the implications if the groups are uneven?

Reviewer #4: This manuscript presents a protocol designed to evaluate and rank the efficacy of several non-pharmacological interventions across different methodological and intervention categories, intending to inform clinical decision-making.

The proposed systematic review and network meta-analysis of randomized controlled trials comparing non-pharmacological interventions with control conditions in individuals with cannabis use disorder is potentially valuable for the fields of addiction medicine, psychiatry, and behavioral medicine. The study is also well positioned to clarify the relative effects of neurotherapeutic modalities in cannabis use disorder.

However, few issues should be addressed to improve the manuscripts clarity and completeness.

First, some acronyms are not defined at their first mention. For example, rTMS and tDCS should be fully written out when they first appear in the manuscript, followed by the abbreviation in parentheses.

Secondly, although this is a protocol, the manuscript would be strengthened if the authors could include any preliminary findings, pilot observations, or feasibility data, where available. If preliminary results are not yet available, the authors should explicitly state this and explain how the protocol will ensure methodological rigor.

Lastly, the authors should briefly discuss possible methodological pitfalls and alternative strategies. For example, they may consider how they would address limited numbers of eligible randomized controlled trials, high heterogeneity across interventions, inconsistent outcome measures, small sample sizes, or disconnected networks in the network meta-analysis. A contingency plan/ alternative approach would improve confidence in the feasibility and robustness of the proposed approach.

Overall, the protocol addresses an important and clinically relevant question in CUD. With improved acronym definition, clearer justification of feasibility, and a brief discussion of alternative methodological approaches in the event of anticipated limitations, the manuscript would be strengthened.

**********

what does this mean?). If published, this will include your full peer review and any attached files.

If you choose “no”, your identity will remain anonymous but your review may still be made public.

**Do you want your identity to be public for this peer review?**  For information about this choice, including consent withdrawal, please see our Privacy Policy

Reviewer #1: **Yes:**  Sura Saad Abdullah

Reviewer #2: No

Reviewer #3: No

Reviewer #4: No

**********

[NOTE: If reviewer comments were submitted as an attachment file, they will be attached to this email and accessible via the submission site. Please log into your account, locate the manuscript record, and check for the action link "View Attachments". If this link does not appear, there are no attachment files.]

To ensure your figures meet our technical requirements, please review our figure guidelines: https://journals.plos.org/plosone/s/figures

You may also use PLOS’s free figure tool, NAAS, to help you prepare publication quality figures: https://journals.plos.org/plosone/s/figures#loc-tools-for-figure-preparation.

NAAS will assess whether your figures meet our technical requirements by comparing each figure against our figure specifications.

# Author comment: Author response to Decision Letter 1

**DOI:** 10.1371/journal.pone.0355941.r002

22 Jul 2026

Subject: Response to Reviewers – Manuscript ID [PONE-D-26-23451]

Dear Editor and Reviewers,

Thank you very much for your time and effort in reviewing our manuscript titled “[Comparative efficacy of non-pharmacological interventions for individuals with cannabis use disorder: a systematic review and network meta-analysis protocol]” (Manuscript ID: [PONE-D-26-23451]). We sincerely appreciate the insightful and constructive comments provided by the reviewers. We have carefully revised the manuscript accordingly and believe that the changes have substantially improved the quality and clarity of the work.

Below, we provide a point-by-point response to all comments. All changes have been highlighted in the revised manuscript.

Response to Reviewer #1

Comment 1: The manuscript refers to an “8-tier methodological framework.” Please provide a concise description or schematic to ensure readers understand how interventions will be categorized and compared.

Response A1: We sincerely thank reviewer for these comments regarding intervention taxonomy. Given overarching categories (such as a generic "8-tier framework" ) risk grouping clinically heterogeneous interventions and compromising the clinical granularity of the network, we have completely overhauled our classification framework. We removed the former 8-tier macro-level framework and replaced it with a highly refined, transparent intervention taxonomy. Modify as follows:

【Methods-Data extraction】“We will extract the core components of each intervention and define them as network nodes, which will be further classified into first-level and second-level nodes. First-level nodes will represent commonly used single interventions as well as frequently applied two- or three-component combinations, thereby preserving network connectivity while avoiding excessive sparsity. These nodes encompassed specific behavioral, motivational, structural, neuromodulatory, and multi-component strategies (e.g., CBT, MET, CM, rTMS, transcranial direct current stimulation, or CBT+MET). Second-level nodes will provide a more granular representation of interventions, enabling sensitivity or subgroup analyses across different outcomes.Where applicable, second-level nodes will be prespecified for specific modalities (e.g., distinguishing abstinence-based CM from attendance-based CM).

Operational variations, including treatment intensity, duration, therapist contact, group versus individual setting, and face-to-face versus digital delivery, were systematically recorded. To prevent network disintegration into disconnected single-study nodes, these trial-level operational features were evaluated as potential sources of heterogeneity using pre-specified network meta-regressions and sensitivity analyses rather than serving as node-defining criteria.”

(Page 11-12)

Comment 2: While primary outcomes are well defined, secondary outcomes include diverse measures (QoL, withdrawal, adverse events). Consider clarifying how heterogeneity across scales will be handled beyond standardization, particularly for QoL instruments.

Response A2: We sincerely thank reviewer for these comments regarding secondary outcomes.

First, we would like to clarify a conceptual ambiguity in our original text: Quality of Life (QoL) is not included as a primary or independent construct in this protocol. Instead, we evaluate cannabis-related problems using validated instruments such as the Cannabis-related Problems Scale(CPS) which captures functional impairments and psychosocial consequences. We have updated the protocol text to explicitly refer to "cannabis-related problems" rather than QoL to avoid conceptual confusion(Page 8).

Second, to address heterogeneity across disparate measurement scales for secondary outcomes (Cannabis-related Problems Scale, withdrawal, adverse events), the specific revisions are as follows�

【Methods-Data synthesis and statistical analysis】“To accommodate potential variations in scoring metrics and assessment tools across included trials, continuous secondary outcomes will be standardized prior to synthesis using Standardized Mean Differences (SMD) with 95% Confidence Intervals (CIs). Scale orientations will be systematically aligned so that higher scores will consistently represent greater symptom severity or functional impairment across all studies. Beyond metric standardization, measurement tools will be audited to ensure they measure clinically comparable constructs. Potential residual heterogeneity originating from scale variations will be systematically evaluated using random-effects models, network meta-regressions, and pre-specified sensitivity analyses omitting outlying assessment instruments.

For specific, narrative, or heterogeneously reported adverse events across primary studies, qualitative descriptive synthesis will be conducted. For standardized and consistently defined binary safety metrics, including the incidence of total adverse events, serious adverse events, or treatment discontinuation attributed to adverse events, quantitative network synthesis will be performed, with pooled effect estimates expressed as Risk Ratios (RRs) with 95% CIs.”

(Page 13-14).

Comment 3: The rationale for excluding quasi-experimental, adaptive, and cross-over designs is reasonable, but a brief explanation of how this exclusion may affect comprehensiveness would strengthen transparency.

Response A3: Thank you for this helpful comment. The specific revisions are as follows�

【Methods-Eligibility criteria】“The quasi-experimental, adaptive, and cross-over designs will be excluded to preserve internal validity and satisfy the transitivity assumption across the network. Specifically, cross-over trials carry substantial risks of persistent carryover effects in cognitive-behavioral and neuromodulatory interventions, while non-randomized designs introduce marked selection bias. Although these strict restrictions minimize methodological heterogeneity, we acknowledge that excluding pragmatic or exploratory study designs may slightly limit the overall comprehensiveness of the retrieved trial landscape. The potential scope and implications of these exclusions will be formally discussed in the final review.”(Page 9)

Comment 4: The current statement indicates data will be available upon request. To align with PLOS ONE’s policy, please clarify whether the minimal dataset will be deposited in a public repository upon completion.

Response A4: We thank the reviewer for this comment and have revised the Data Availability Statement in the manuscript to ensure full compliance with PLOS ONE’s policy, as detailed below: 【DATA AVAILABILITY STATEMENT】“All relevant data supporting the planned systematic review and network meta-analysis will be made publicly available upon completion of the study. The minimal dataset, including standardized data extraction sheets, summary outcome data, and analytic scripts, will be deposited in a public repository such as the Open Science Framework (OSF) with a persistent DOI. No primary individual patient data are generated for this protocol.”(Page 24)

Comment 5: Minor grammatical and stylistic edits would improve readability (e.g., streamline long sentences in the introduction and methods).

This protocol is methodologically sound and addresses a critical gap in comparative evidence for CUD interventions. With minor clarifications and improvements in reporting, it will provide a strong foundation for the planned systematic review and network meta-analysis. I recommend minor revisions to enhance clarity and compliance with journal standards.

Response A5: We sincerely thank the reviewer for the encouraging summary and constructive feedback.

In accordance with your recommendation, we have thoroughly proofread and revised the manuscript. Specifically, we streamlined complex and lengthy sentences across the Introduction and Methods sections, corrected minor grammatical inconsistencies, and enhanced sentence transitions to improve overall readability and conciseness. All revisions have been carefully made to strictly align with journal formatting and reporting standards without altering the core methodological substance.

Response to Reviewer #2

Comment 1: The population definition is too broad for cannabis use disorder

The protocol claims to study individuals with CUD, but the eligibility criteria allow inclusion of participants who used cannabis in the past month and had at least one cannabis-related problem. That is not equivalent to a formal diagnosis of CUD, cannabis dependence, or cannabis abuse. This could introduce major clinical heterogeneity and weaken the applicability of the findings.

The authors should separate formally diagnosed CUD from broader “problematic cannabis use” populations, or at least plan subgroup or sensitivity analyses by diagnostic certainty.

Response B1: We sincerely thank the reviewer for this helpful comment. The specific revisions are as follows�

【Methods-Eligibility criteria】“To account for clinical heterogeneity, participants will be classified into formally diagnosed CUD cases (DSM or ICD criteria) and screening-identified problematic cannabis users (e.g., recruited from detoxification or emergency settings, or reporting past-month use with baseline problems).The primary analysis will synthesize all eligible populations combined. Pre-specified subgroup and sensitivity analyses will evaluate whether diagnostic certainty alters treatment estimates. If significant heterogeneity or subgroup differences are identified, findings will be stratified and reported separately by diagnostic certainty. Otherwise, the overall pooled synthesis will serve as the primary output.”

(Page 7)

Comment 2: Age criteria are unclear and potentially inconsistent

The table defines adults as age ≥16 years, and later the manuscript discusses adolescents and young adults. In many jurisdictions, 16-year-olds are adolescents, not adults. The authors also plan a sensitivity analysis excluding adolescent trials, but the main inclusion criteria already mix adolescents and adults.

The authors should clearly define age groups and decide whether the review is about adults, adolescents, or both. If both are included, age should be treated as a major effect modifier in the NMA.

Response B2: We sincerely thank the reviewer for this helpful comment. The specific revisions are as follows�

【Methods-Eligibility criteria】“Age groups will be explicitly categorized as adolescents (aged 12–17 years) and adults (aged ≥18 years). To address potential clinical heterogeneity driven by age differences, age will be evaluated as a effect modifier across primary outcomes. Network meta-regressions incorporating trial-level mean age will be conducted to detect potential effect modification. Additionally, pre-specified subgroup analyses stratified by age category (adolescents vs. adults) and sensitivity analyses excluding adolescent trials will be performed to ensure the validity of the final network rankings.”(Page 7)

Comment 3: Outcomes are not sufficiently well defined

The primary outcomes are confusing. The abstract lists efficacy, acceptability, and cannabis-use days as primary outcomes. The methods describe efficacy as both abstinence and days of cannabis use. The table lists point abstinence, cannabis-use days, and retention. Secondary outcomes then include cannabis-use frequency over the past 30 days, which overlaps with cannabis-use days.

The authors should define one clear hierarchy of outcomes. For example:

Primary benefit outcome: biochemically verified abstinence at end of treatment.

Primary harm/acceptability outcome: all-cause dropout or treatment discontinuation.

Secondary outcomes: days of cannabis use, cannabis-related problems, dependence severity, craving, withdrawal, mood symptoms, adverse events.

Response B3: We sincerely thank the reviewer for this helpful comment. The specific revisions are as follows�

【Methods-Eligibility criteria】

“Primary outcomes will evaluate intervention benefit, acceptability, and safety.

1)Primary benefit outcomes will include: (a) biochemically verified cannabis abstinence at the end of treatment, and (b) total days of cannabis use over the past 30 days (self-reported).

2)Primary Acceptability Outcome: Treatment retention, defined as the proportion of randomized participants who complete at least one session during the final week of treatment, based on an intention-to-treat approach. Additionally, retention at follow-up timepoints will be calculated as the proportion of randomized participants completing follow-up assessments without dropout.

3)Primary Safety/Harm Outcome: Overall incidence of adverse events (and serious adverse events) attributed to the intervention.

Secondary outcomes will capture consumption intensity and broader clinical impacts, including:

1)Quantity of cannabis use: Measured as the number of joints or grams consumed over the past 30 days.

2)Cannabis-related problems: Assessed via validated instruments such as the Cannabis Problems Scale (CPS).

3)Withdrawal and psychopathology: Withdrawal severity and associated psychological symptoms (including dependence, anxiety, and depression) ”

(Page 8)

Comment 4: Intervention node classification is underdeveloped

The eight intervention categories are potentially useful, but the node-building rules are not precise enough. For example, “combined interventions” may include CBT plus MET, CBT plus CM, MET plus CM, family therapy plus CM, or psychosocial intervention plus neuromodulation. These are clinically very different and may not be valid as one node.

The authors should provide explicit rules for classifying multicomponent interventions, including how they will handle intensity, duration, therapist contact, delivery mode, group versus individual format, digital versus face-to-face delivery, and co-interventions.

Response B4: We sincerely thank reviewer for these comments regarding intervention node classification. Given overarching categories (such as a generic "8-tier framework" ) risk grouping clinically heterogeneous interventions and compromising the clinical granularity of the network, we have completely overhauled our classification framework. We removed the former 8-tier macro-level framework and replaced it with a highly refined, transparent intervention taxonomy. Modify as follows:

【Methods-Data extraction】“We will extract the core components of each intervention and define them as network nodes, which will be further classified into first-level and second-level nodes. First-level nodes will represent commonly used single interventions as well as frequently applied two- or three-component combinations, thereby preserving network connectivity while avoiding excessive sparsity. These nodes encompassed specific behavioral, motivational, structural, neuromodulatory, and multi-component strategies (e.g., CBT, MET, CM, rTMS, transcranial direct current stimulation, CBT+MET, or CBT+CM). Second-level nodes will provide a more granular representation of interventions, enabling sensitivity or subgroup analyses across different outcomes.Where applicable, second-level nodes will be prespecified for specific modalities (e.g., distinguishing abstinence-based CM from attendance-based CM).

Operational variations, including treatment intensity, duration, therapist contact, group versus individual setting, and face-to-face versus digital delivery, were systematically recorded. To prevent network disintegration into disconnected single-study nodes, these trial-level operational features were evaluated as potential sources of heterogeneity using pre-specified network meta-regressions and sensitivity analyses rather than serving as node-defining criteria.”

(Page 11-12)

Comment 5: Statistical methods contain internal inconsistencies

The statistical section needs substantial revision. The authors describe a frequentist NMA but refer to “credible intervals,” which are usually Bayesian. They also mention weighted least squares with REML and then say they will apply the DerSimonian-Laird random-effects model. REML and DerSimonian-Laird are different estimators and should not be presented as the same approach.

Response B5: We sincerely thank the reviewer for identifying these statistical inconsistencies. We have fully re

Attachment Submitted filename: Response to Reviewers.docx

# Editor report: Decision Letter 1

**DOI:** 10.1371/journal.pone.0355941.r003

**Authors:** Alberto Sá Filho

29 Jul 2026

Comparative efficacy of non-pharmacological interventions for individuals with cannabis use disorder: a systematic review and network meta-analysis protocol

PONE-D-26-23451R1

Dear Dr. Lu

We’re pleased to inform you that your manuscript has been judged scientifically suitable for publication and will be formally accepted for publication once it meets all outstanding technical requirements.

Within one week, you’ll receive an e-mail detailing the required amendments. When these have been addressed, you’ll receive a formal acceptance letter and your manuscript will be scheduled for publication.

An invoice will be generated when your article is formally accepted. Please note, if your institution has a publishing partnership with PLOS and your article meets the relevant criteria, all or part of your publication costs will be covered. Please make sure your user information is up-to-date by logging into Editorial Manager at Editorial Manager® and clicking the ‘Update My Information' link at the top of the page. For questions related to billing, please contact billing support.

If your institution or institutions have a press office, please notify them about your upcoming paper to help maximize its impact. If they’ll be preparing press materials, please inform our press team as soon as possible -- no later than 48 hours after receiving the formal acceptance. Your manuscript will remain under strict press embargo until 2 pm Eastern Time on the date of publication. For more information, please contact onepress@plos.org.

Kind regards,

Alberto Souza Sá Filho, Ph.D

Academic Editor

PLOS One

# Editor report: Acceptance letter

**DOI:** 10.1371/journal.pone.0355941.r004

**Authors:** Alberto Sá Filho

PONE-D-26-23451R1

PLOS One

Dear Dr. Lu,

I'm pleased to inform you that your manuscript has been deemed suitable for publication in PLOS One. Congratulations! Your manuscript is now being handed over to our production team.

At this stage, our production department will prepare your paper for publication. This includes ensuring the following:

* All references, tables, and figures are properly cited

* All relevant supporting information is included in the manuscript submission,

* There are no issues that prevent the paper from being properly typeset

You will receive further instructions from the production team, including instructions on how to review your proof when it is ready. Please keep in mind that we are working through a large volume of accepted articles, so please give us a few days to review your paper and let you know the next and final steps.

Lastly, if your institution or institutions have a press office, please let them know about your upcoming paper now to help maximize its impact. If they'll be preparing press materials, please inform our press team within the next 48 hours. Your manuscript will remain under strict press embargo until 2 pm Eastern Time on the date of publication. For more information, please contact onepress@plos.org.

You will receive an invoice from PLOS for your publication fee after your manuscript has reached the completed accept phase. If you receive an email requesting payment before acceptance or for any other service, this may be a phishing scheme. Learn how to identify phishing emails and protect your accounts at https://explore.plos.org/phishing.

If we can help with anything else, please email us at customercare@plos.org.

Thank you for submitting your work to PLOS One and supporting open access.

Kind regards,

PLOS ONE Editorial Office Staff

on behalf of

Dr. Alberto Souza Sá Filho

Academic Editor

PLOS One
